# Histone methyltransferase Smyd3 is a new regulator for vascular senescence

**DOI:** 10.1111/acel.13212

**Published:** 2020-08-11

**Authors:** Di Yang, Gang Wei, Fen Long, Hongbo Nie, Xiaoli Tian, Lefeng Qu, ShuangXi Wang, Peng Li, Yue Qiu, Yang Wang, Wanjin Hong, Ting Ni, Xinhua Liu, Yi Zhun Zhu

**Affiliations:** ^1^ Department of Pharmacology Human Phenome Institute School of Pharmacy Fudan University Shanghai China; ^2^ State Key Laboratory of Quality Research in Chinese Medicine and School of Pharmacy Macau University of Science and Technology Taipa China; ^3^ State Key Laboratory of Genetic Engineering & MOE Key Laboratory of Contemporary Anthropology Collaborative Innovation Center of Genetics and Development Human Phenome Institute School of Life Sciences and Huashan Hospital Fudan University Shanghai China; ^4^ Human Aging Research Institute School of Life Sciences Nanchang University Nanchang China; ^5^ Department of Vascular Surgery Changzheng Hospital Second Military Medical University Shanghai China; ^6^ The Key Laboratory of Cardiovascular Remodeling and Function Research Qilu Hospital Shandong University Jinan China; ^7^ College of Pharmacy Xinxiang Medical University Xinxiang China; ^8^ Institute of Molecular and Cellular Biology Singapore City Singapore

**Keywords:** angiotensin II, endothelial cell, p21, Smyd3, vascular senescence

## Abstract

Endothelial cell senescence is one of the main risk factors contributing to vascular diseases. As increasing number of “epigenetic drugs” entering clinical trials, understanding the mechanism of epigenetic regulation in vascular aging has significant implications in finding targets to cure vascular diseases. However, the epigenetic regulation of endothelial senescence remains unclear. Based on the findings that increased protein level of histone H3 lysine 4 (H3K4) methyltransferase Smyd3 and elevated H3K4me3 modification happened in angiotensin II (Ang II)‐induced senescence in rat endothelial cells, we are curious about whether and how Smyd3 can regulate endothelial senescence. We found that an increase of Smyd3 alone promoted senescence‐associated phenotypes, while knockdown of Smyd3 blocked senescence in endothelial cells. Furthermore, Smyd3‐specific inhibitor reversed vascular senescence‐associated phenotypes at cellular level. Importantly, Ang II‐induced vascular senescence can be greatly alleviated in Smyd3 knockout (KO) mice and those treated with Smyd3 inhibitor. Mechanistically, Smyd3 directly bound to the promoter region of *Cdkn1a* (coding for p21), then caused its increased H3K4me3 level and elevated gene expression, and ultimately gave rise to senescence‐associated phenotypes. Intriguingly, Smyd3‐mediated p21 upregulated expression also exists in human tissues of vascular disease, indicating it is probably an evolutionarily conserved mechanism in regulating vascular senescence. Thus, Smyd3 can act as a novel factor regulating endothelial senescence through transcriptionally promoting p21 expression. Blocking the Smyd3‐p21 signaling axis may also have potential medical implications in treating diseases related to vascular aging.

## Graphical abstract

Younger cell has lower levels of Smyd3 protein and H3K4me3 at the promoter region of p21 gene, with the result of low abundance of p21 protein. In endothelial senescence systems including Ang II‐induced RAECs, Ang II‐infused mice, replicative senescence, hypertensive rat model, and atherosclerotic human tissues, Smyd3 protein level was increased, leading to higher accumulation of H3K4me3 at the promoter of p21 gene, which further results in higher expression of p21, and finally causes senescence‐associated phenotypes.

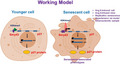

## 1 INTRODUCTION

Aging is accompanied by the progressive decline of proper organ functions and gradual body caducity. Aging can have profound effects on vasculature, giving rise to multiple senescence‐associated phenotypes, which in all is termed as vascular aging. Vascular aging is the main risk factor contributing to vascular dysfunction and the progression of vascular diseases. As blood vessels age, they accumulate increasing number of senescent vascular cells, among of which, senescent endothelial cells (ECs) (usually with dysfunction) were seen as a major hallmark of vascular disease (Donato, Morgan, Walker, & Lesniewski, 2015). With age advancing, accumulated senescence‐associated damage to ECs can lead to endothelial dysfunction, which in turn induces a proinflammatory state and further exacerbates the cacoethic effect to ECs and even promotes vascular diseases (such as atherosclerosis, hypertension, and stroke) (Abbas et al., 2017; Blann, 2003). As to now, vascular endothelial cell senescence has been increasingly recognized as an important factor contributing to the pathogenesis of cardiovascular diseases (Dorado & Andres, 2017; Fuster & Andres, 2006; Minamino & Komuro, 2007; Tian & Li, 2014). Eliminating senescent cells can attenuate age‐related deterioration and extend healthy life span (Baker et al., 2016). These findings highlight the biological and biomedical importance of understanding the factors regulating endothelial cell senescence.

Senescence of endothelial cell has the regular features of normal cell senescence, such as irreversible growth arrest, elevated expression or activation of p53, increased expression of p21^CIP1/WAF1^ and p16^INK4a^ (the two cell cycle regulators), and increased senescence‐associated β galactosidase activity (SA‐β‐Gal) (Krishnamurthty et al., 2004) (Dimri et al., 1995). Senescence in endothelial cells also features with senescent‐associated secretory phenotype (SASP), which is characterized by high level of matrix metalloproteinase (MMPs) and proinflammatory cytokines (van Deursen, 2014). These molecular events above can be looked as important indicators of vascular dysfunction.

Many differences in senescent cells compared to normal cells may underlie the causes contributing to senescent phenotypes (Kim et al., 2013), among of which, dramatic epigenetic alterations have been realized as a new trigger causes of senescent cells (Abad et al., 2011). Epigenetic alterations (such as DNA methylation and histone modifications) have been viewed as one of the hallmarks of aging (Lopez‐Otin, Blasco, Partridge, Serrano, & Kroemer, 2013), it can also be an important contributor in regulating cellular function during aging. Histones are subjected to a wide variety of posttranslational modifications (PTM) that can have multiple impacts on the global structure of the chromatin, which in turn influences gene expression, genome stability, and replication (Petryk et al., 2018). This array of histone modifications orchestrates to regulate various biological processes including aging and senescence. Imbalance of activating and repressive histone modifications has been reported to occur in senescence (Paluvai, Di Giorgio, & Brancolini, 2020). Histone modifications can be under the complex regulation of variety of modifications, including histone methylation and histone acetylation (Wang, Yuan, & Xie, 2018). Smyd3, a SET (Su) and MYND (myeloid‐Nervy‐DEAF‐1) domain‐containing protein 3, catalyzes both dimethylation and trimethylation of histone H3 at lysine 4 (H3K4). The resulting H3K4me2 and H3K4me3 near the promoter regions of target genes usually serve as transcriptional activation markers and can induce oncogene activation and promote cell growth (Giakountis, Moulos, Sarris, Hatzis, & Talianidis, 2017; Hamamoto et al., 2004b). Of note, Smyd3 can increase the H3K4me3 level of MMP9 promoter and cell migration rate in cancer cells (Cock‐Rada et al., 2012). Besides its H3K4 methyltransferase activity, Smyd3 can also catalyze the methylation of H4K5 and H2A.Z, which function in cancer cell growth and other related phenotypes (Tsai et al., 2016). Moreover, Smyd3 has the capacity to modify non‐histone proteins VEGFR (Kunizaki et al., 2007) and MAP3K2 to promote metastasis and Ras/Raf/MEK/ERK signaling (Mazur et al., 2014) in cancer development. Together, Smyd3 can regulate various cellular phenotypes through its histone‐dependent (H3K4, H4K5, and H2A.Z) and histone‐independent capacity to modify proteins.

Previous reports showed increased expression of Smyd3 in multiple cancer types and Smyd3 overexpression could contribute to cancer‐associated phenotypes through diverse mechanisms (Bottino, Peserico, Simone, & Caretti, 2020). In addition, Ang II‐treated rat primary aorta endothelial cells (RAECs) has been widely used as an ideal model for vascular aging study (Yoshimoto et al., 2005). Here, we found Smyd3 exhibited increased expression in Ang II‐induced endothelial cell senescence. In addition, Smyd3 accumulation led to senescence‐related phenotypes in endothelial cells. Furthermore, inhibition of Smyd3 attenuated senescence‐associated phenotypes both in vitro and in vivo. Intriguingly, the senescence‐accompanied accumulation of Smyd3 (with H3K4 methyltransferase activity) was echoed by increased H3K4me3 level at p21 promoter, which could in turn regulate endothelial cellular senescence. Thus, targeting Smyd3 might be a promising approach to ameliorating the effects of age‐related vascular dysfunction. We revealed for the first time that manipulation of Smyd3 can contribute to cellular senescence but not cancer in endothelial cells.

## 2 RESULTS

### 2.1 Smyd3 is upregulated in Ang II‐induced senescence in both cell and mice models

**Figure 1:**
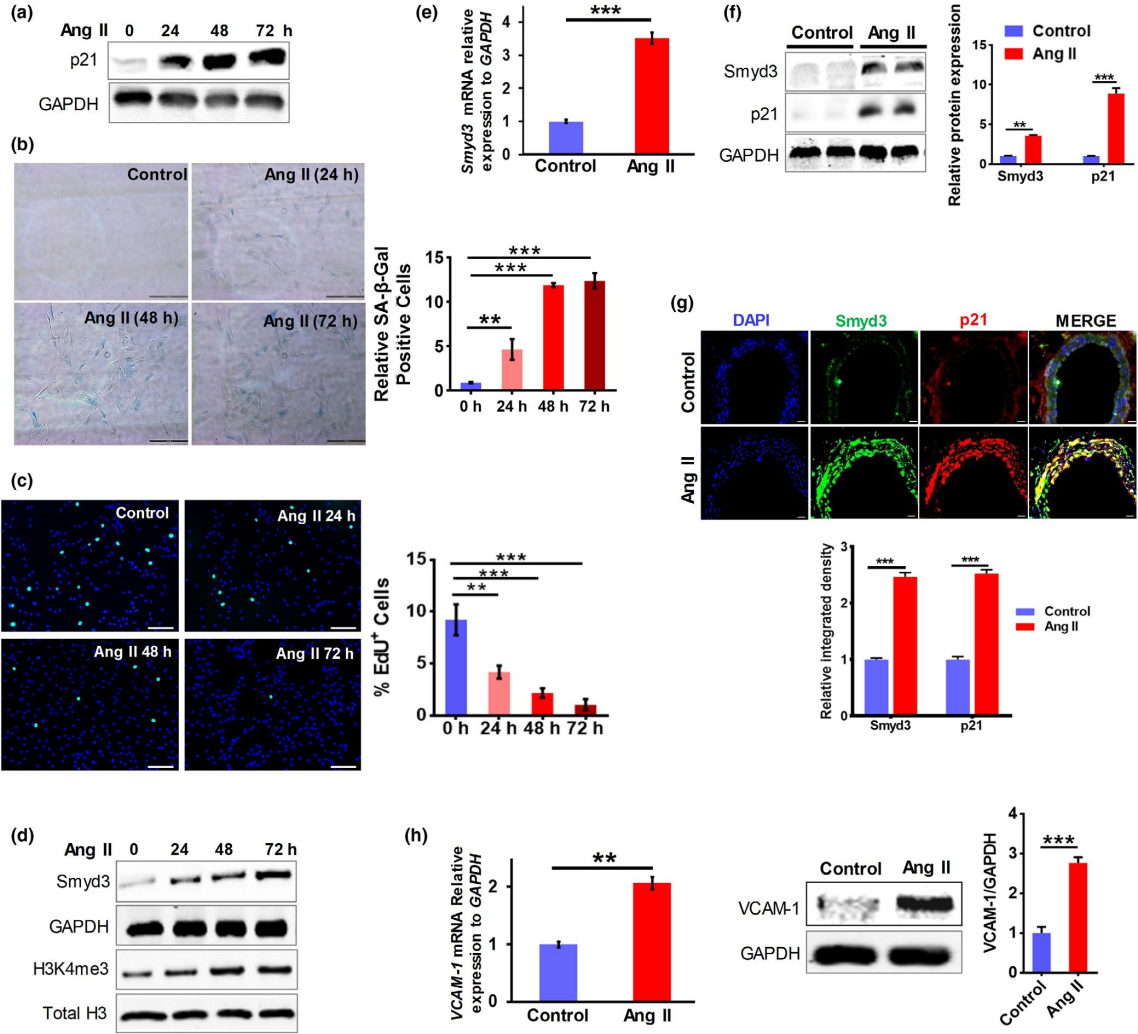
Upregulation of Smyd3 in Ang II‐induced vascular senescence. (a) Increased expression of p21 at protein level in Ang II‐induced RAECs assessed by Western blot. GAPDH serves as the loading control, blots shown are representative of data from at least three different replicates. (b) Left: Representative images of SA‐β‐Gal staining of RAECs. Right: the percentage of the SA‐β‐Gal positive cells, shown as mean ± SEM, ^**^ *p* < 0.01, ^***^ *p* < 0.001, (*n* ≥ 3). (c) Left: Representative images of EdU incorporation assay of RAECs before and after Ang II treatment. Right: Quantification of percent EdU^+^ cells, shown as mean ± SEM, ^**^ *p* < 0.01, ^***^ *p* < 0.001, (*n* ≥ 3). (d) Increased protein expression of Smyd3 and H3K4me3 in Ang II‐induced RAECs assessed by Western blot. GAPDH and total H3 serve as the loading control, blots shown are representative of data from at least three different replicates. (e) mRNA level of *Smyd3* in aortic great vessels of Ang II‐infused mice compared to control mice evaluated by qRT‐PCR. ^***^ *p* < 0.001, *n* = 6. (f) Quantification of Smyd3 and p21 protein levels in aortic great vessels of Ang II‐infused mice compared to control mice assayed by Western blot, shown as mean ± SEM, ^**^ *p* < 0.01, ^***^ *p* < 0.001, *n* = 6. (g) Immunofluorescence double staining and quantification of Smyd3 and p21 in aortic great vessels of Ang II‐infused and control mice. “DAPI” represents DAPI staining of nuclei (DNA) throughout the manuscript. Constant infusion with Ang II led to increased integrated density of Smyd3 and p21 staining. The results are presented as the means ± SEM, ^***^ *p* < 0.001, *n* = 6. (h) mRNA and protein level of VCAM‐1 in aortic great vessels of Ang II‐infused mice compared to control mice, as assayed by qRT‐PCR and Western blot, respectively, shown as mean ± SEM, ^**^ *p* < 0.01, ^***^ *p* < 0.001, *n* = 6

Ang II‐induced RAECs senescence model was first established. To confirm that Ang II treatment does induce senescence‐related phenotypes in RAECs, we examined the expression of multiple senescence markers. The results showed that Ang II‐treated RAECs exhibited increased protein level of p21, a cyclin‐dependent kinase inhibitor and a well‐know senescence marker (Figure 1a). Moreover, the significant increase in the staining of SA‐β‐Gal (Figure 1b) and decrease in EdU (5‐Ethynyl‐2′‐deoxyuridine) incorporation (Figure 1c) were observed in Ang II‐treated cells. In addition, more extensive analysis on other senescence markers (including p16, VCAM‐1, IL‐6, iNOS, p‐ATM, p‐Chk2, and γ‐H2AX) also demonstrated that Ang II‐treated RAECs exhibited characteristic phenotypes of vascular cell senescence (Figure S1). Interestingly, Smyd3 showed a continuously increased expression during Ang II‐induced senescence in RAECs (Figure 1d). Consistent with the H3K4 methyltransferase activity of Smyd3 and its elevated expression, senescent cells exhibited a progressive increase in trimethylation of H3K4 (H3K4me3) abundance while the total histone H3 levels kept unchanged (Figure 1d). In contrast, other histone modifications including H3K4me1, H3K27me3, H3K27ac, H3K36me2, and H3K9me3 did not show significant increase upon Ang II induction (Figure S2). These results above combined to indicate that higher Smyd3 level may be associated with Ang II‐induced vascular aging using RAECs as a cell model.

To examine whether such association also exists in vivo, mice were subjected to a 28‐day infusion of saline or Ang II, and aortic great vessels were dissected for series of assays. Strikingly, Smyd3 expression dramatically increased in aortic great vessels of Ang II‐infused mice (Figure 1e,f). Moreover, p21, the well‐known senescence marker, also showed significantly increased expression in aortic vessels of Ang II‐infused mice (Figure 1f), similar to that in Ang II‐treated RAECs (Figure 1a). Interestingly, Smyd3 and p21 both showed significantly increased expression in the vascular wall (including the inner wall where endothelial cells locate) of Ang II‐infused mice, as detected by immunofluorescence double staining (Figure 1g). Furthermore, VCAM‐1, a senescence‐associated secretory protein, also showed significantly increased expression in Ang II‐infused mice compared with control ones (Figure 1h). All these results above combined to support the idea that Smyd3 is associated with vascular aging in both cellular and animal model.

### 2.2 Smyd3 directly regulates Ang II‐induced senescence in RAECs

**Figure 2:**
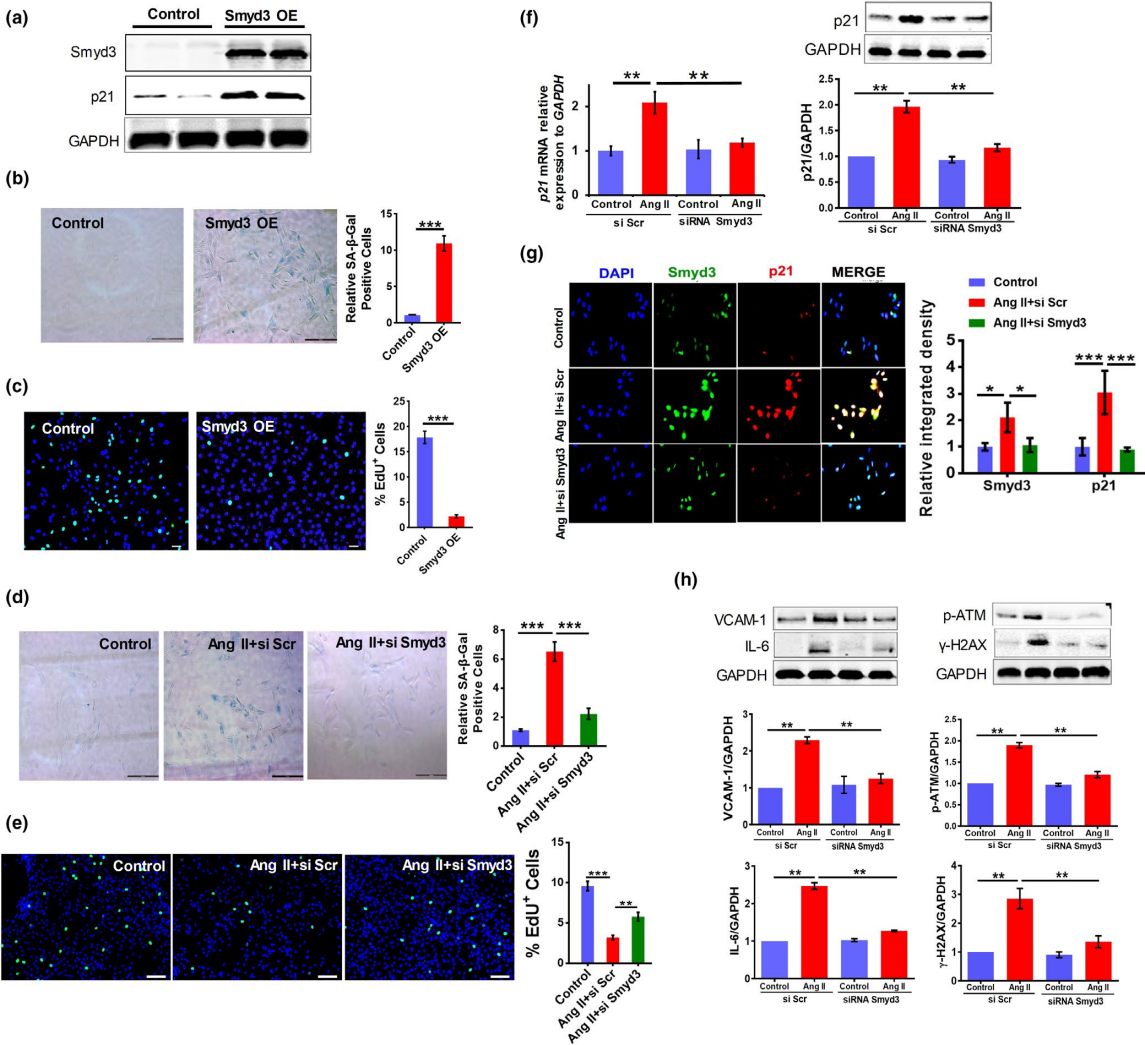
Smyd3 overexpression promotes senescence and Smyd3 knockdown mitigates vascular senescence in Ang II‐induced RAECs. (a–c) Smyd3 overexpression promotes RAECs senescence. Overexpression (OE) of Smyd3 was confirmed by Western blot and p21 level increases in Smyd3 overexpressing cells, all blots shown are representative images from at least three replicates (a). SA‐β‐Gal staining with the percentage of the SA‐β‐Gal positive cells (b) and EdU incorporation assay with the quantification of percent EdU^+^ cells (c) of RAECs overexpressing Smyd3, shown as mean ± SEM, ^***^ *p* < 0.001, (*n* ≥ 3). (d–h) Smyd3 knockdown (KD) reverses senescence in Ang II‐induced RAECs. SA‐β‐Gal staining with the percentage of the SA‐β‐Gal positive cells (d) and EdU incorporation assay with the quantification of percent EdU^+^ cells (e), shown as mean ± SEM, ^**^ *p* < 0.01, ^***^ *p* < 0.001, (*n* ≥ 3); The expression changes of p21 at mRNA and protein level, data were derived from at least three biological replicates, ^**^ *p* < 0.01 (f); Immunofluorescence double staining of Smyd3 and p21, and quantification results are presented as the means ± SEM; ^*^ *p* < 0.05, ^***^ *p* < 0.001, *n* = 6. (g) and expression of senescence‐associated markers (VCAM‐1, IL‐6, p‐ATM, and γ‐H2AX) assayed by Western blot (h) for RAECs treated with Ang II (Ang II +si Scr), those of Smyd3‐KD and treated with Ang II (Ang II +si Smyd3), and control RAECs. GAPDH serves as internal control, ^**^ *p* < 0.01, data were derived from at least three biological replicates

Having discovered that Smyd3 is associated with Ang II‐induced senescence and vascular aging, we are still curious whether Smyd3 can directly regulate senescence at cellular level. To address this question, we first overexpressed Smyd3 in RAECs and then examined senescence‐associated phenotypes. The results showed that Smyd3‐overexpressed RAECs showed apparent senescence‐related phenotypes, including significantly elevated expression of the senescence marker p21 (Figure 2a), increased percentage of cells with positive SA‐β‐Gal staining (Figure 2b) and decreased EdU incorporation (Figure 2c). These above evidences support that higher level of Smyd3 alone can promote cellular senescence.

To further confirm the role of Smyd3 in regulating vascular cell senescence, we next downregulated Smyd3 expression in RAECs using small interfering RNA (siRNA) (Figure S3). Both the Smyd3 knockdown (Smyd3‐KD) cells and the control ones were treated with Ang II, and then the senescence‐associated phenotypes were examined. Interestingly, Smyd3‐KD cells showed decreased Ang II‐induced SA‐β‐Gal activity (Figure 2d) and increased EdU incorporation (Figure 2e). In addition, Smyd3‐KD dramatically downregulated Ang II‐induced p21 increase at both mRNA and protein level in RAECs (Figure 2f). Moreover, Ang II‐induced co‐expression of Smyd3 and p21 disappeared in Smyd3‐KD RAECs, as assayed by immunofluorescence double staining (Figure 2g). Besides, Smyd3‐KD decreased the expression of senescence‐associated markers in Ang II‐treated RAECs (Figure 2h). Furthermore, impaired nitric oxide (NO)‐mediated endothelium‐dependent dilation is a major antecedent to numerous chronic clinical disorders of aging (Gimbrone & Garcia‐Cardena, 2016), we showed that the level of NO decreased whereas the level of superoxide increased after the Ang II treatment, which could be reversed by knockdown of Smyd3 with either siRNA or inhibition with small molecule inhibitor EPZ031686 (Figure S4). These results demonstrate that Smyd3 can directly regulate Ang II‐induced vascular senescence.

### 2.3 Smyd3 expression is mediated by p‐Smad3 in Ang II‐induced RAEC cells

**Figure 3:**
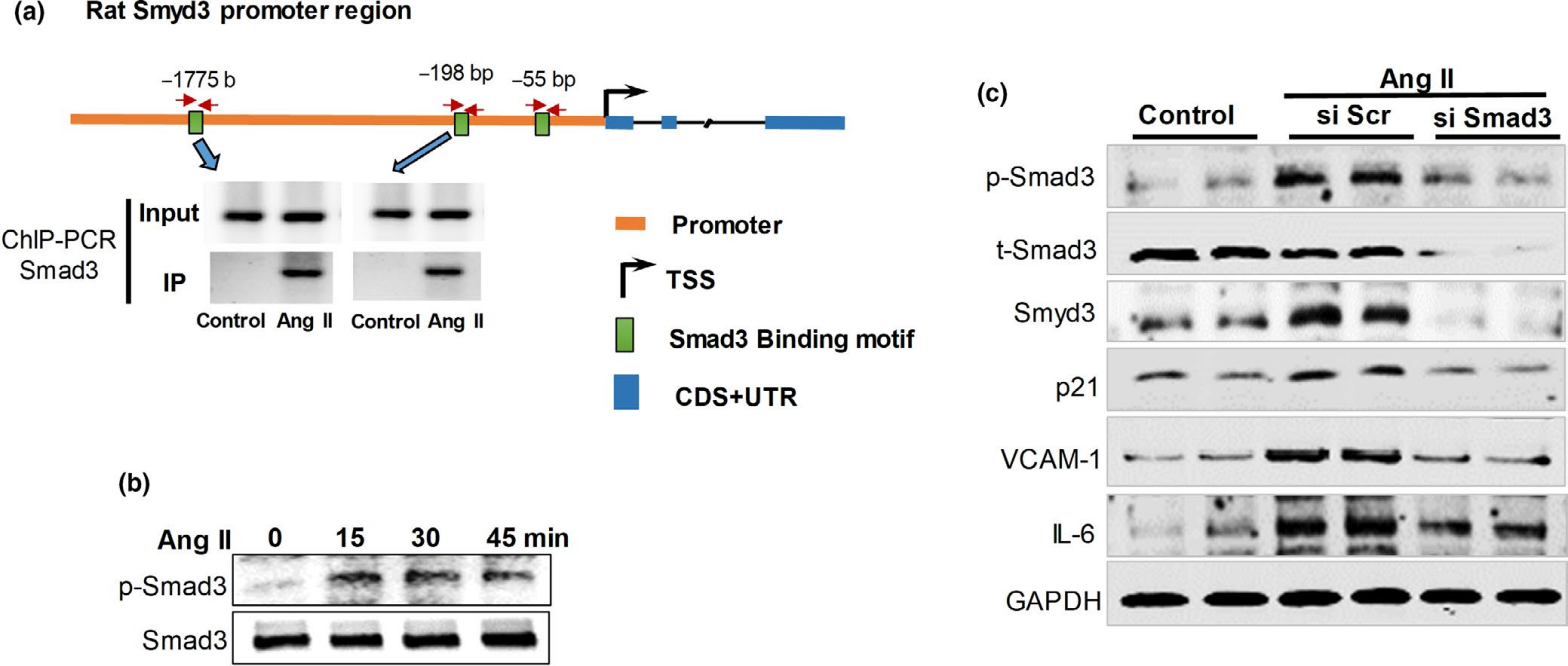
p‐Smad3 regulates Smyd3 expression in RAEC cells. (a) The promoter region of rat Smyd3 gene contains three Smad3 binding motifs, the binding of Smad3 to Smyd3 promoter significantly increased in Ang II‐treated RAECs assayed by ChIP‐PCR with Smad3 antibody. (b) Protein abundance of p‐Smad3 and Smad3 in RAECs treated with Ang II evaluated by Western blot. (c) Protein abundance of p‐Smad3, t‐Smad3, Smyd3, p21, VCAM‐1, IL‐6 in Smad3 knockdown RAECs (si Smad3) and control RAECs (scramble siRNA [si Scr]) before and after Ang II treatment, GAPDH serves as the internal control, blots shown are representative of data from at least three different replicates

Smad3, a DNA‐binding protein and a transcription factor of SMAD family, has been found having three binding sites within 1.1 kb of the Smyd3 promoter region in human iTreg cells, and the one located in the core promoter (104 bp from TSS [transcription start site]) is evolutionarily conserved (Nagata et al., 2015). Furthermore, iTreg cells transfected with siRNA targeting Smad3 displayed significant decreases in Smyd3 along with decreased accumulation of H3K4me3 (Nagata et al., 2015). Thus, we examined whether Smad3 could also be a potential upstream transcription factor of Smyd3 in RAECs. It was interesting to find that three Smad3 binding sites existed within 2 kilobase (kb) upstream Smyd3 coding region in rat (Figure 3a), two of which located within the core promoter region (−198 to −55 bp upstream the TSS) (Figure 3a). Of note, abundance of phosphorylated Smad3 (p‐Smad3) significantly increased upon Ang II treatment (Figure 3b), indicating that Ang II probably activates Smad3 pathways through phosphorylation activation. Interestingly, RAECs treated with Ang II showed enhanced Smad3 binding at the Smyd3 promoter region compared with control cells determined by chromatin immunoprecipitation coupled with polymerase chain reaction (ChIP‐PCR) assay (Figure 3a, the quantification was shown in Figure S5). To validate the regulatory role of Smad3 on Smyd3, we knock down Smad3 with siRNA in RAECs. The result showed that depletion of Smad3 led to significant decrease in Smyd3 and p‐Smad3 at protein level, accompanied by reduced expression of vascular senescence markers including p21, VCAM‐1, and IL‐6 (Figure 3c). These results suggest that upregulated expression of Smyd3 in Ang II‐induced cells is mediated by Smad3‐dependent mechanism.

### 2.4 Smyd3 promotes cellular senescence by binding to the promoter region of p21 gene

We next investigated the underlying mechanism how Smyd3 promotes vascular senescence. To figure out the direct downstream targets of Smyd3, we performed chromatin immunoprecipitation coupled with deep sequencing (ChIP‐seq) and RNA sequencing (RNA‐seq) on Ang II‐induced and control RAEC cells. We found globally increased ChIP‐seq signal of H3K4me3 near transcription start site (TSS) in Ang II‐induced cells (Figure S6A). Interestingly, MAPK signaling pathway and cell cycle are the most significantly enriched pathways for genes with increased H3K4me3 and those with decreased H3K4me3, respectively (Figure S6B–C). RNA‐seq data were then included to analyze the correlation between H3K4me3 level and expression level. Consistent with the previous reports that H3K4me3 abundance at the promoter region often positively correlates with gene expression levels (Shao, Zhang, Yuan, Orkin, & Waxman, 2012; Shi et al., 2006), we found more genes tended to be upregulated expression in genes with increased H3K4me3, while the contrary trend existed in genes with deceased level of H3K4me3 (Figure S7). RNA‐seq results also showed that ECM–receptor interaction, cytokine–cytokine receptor interaction, and hypertrophic cardiomyopathy were the several top enriched pathways for genes with upregulated expression upon Ang II treatment. While cell cycle, DNA replication and mismatch repair were the several top enriched pathways for downregulated genes (Figure S8), in line with the reduced proliferation rate of Ang II‐treated cells.

**Figure 4:**
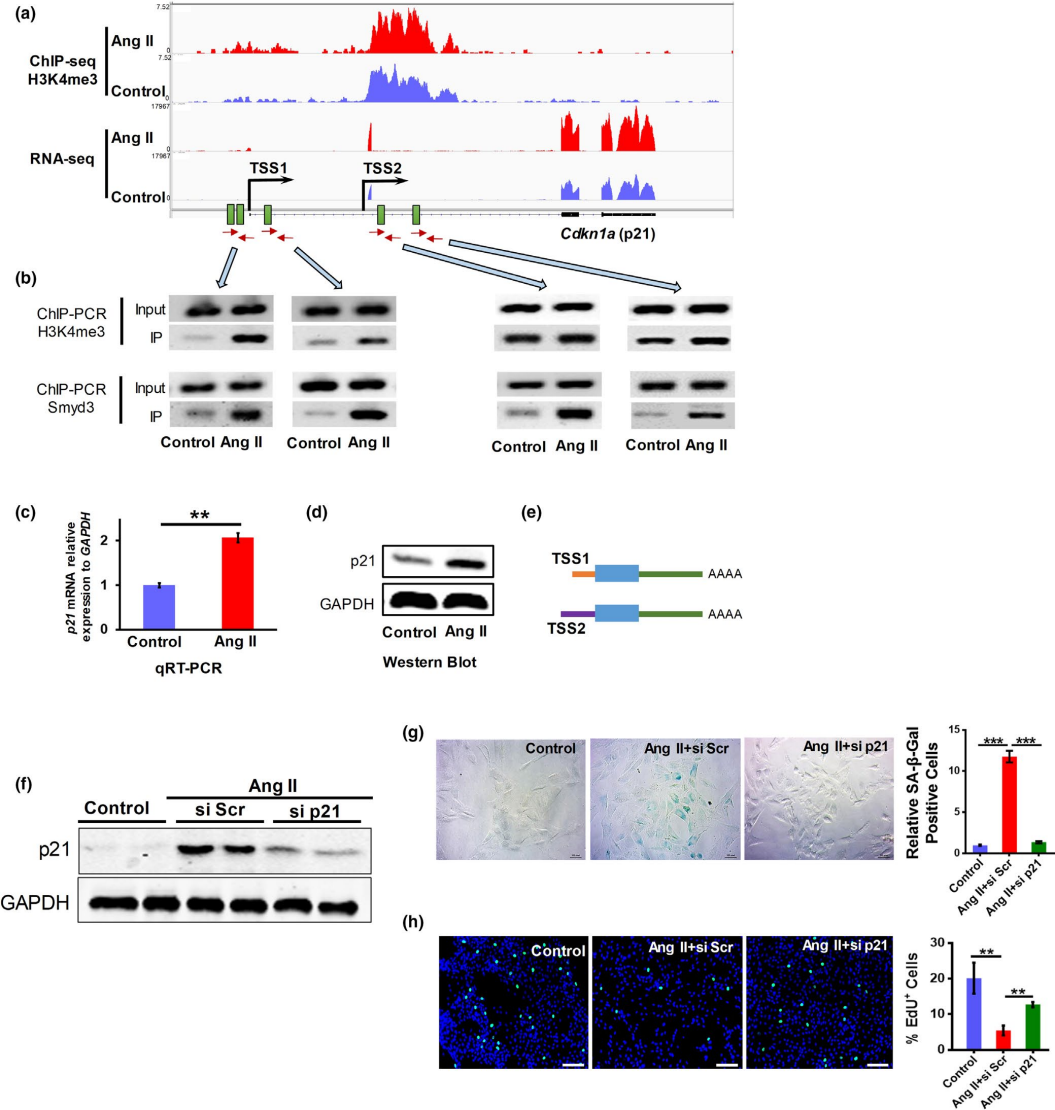
Binding of Smyd3 to both promoters of p21 transcript variants leads to increased H3K4me3 level and elevated gene expression. (a) ChIP‐seq and RNA‐seq tracks illustrating H3K4me3 and expression level of p21 gene in Ang II‐induced (Ang II, 48 h) and control RAECs, as shown in Integrative Genomics Viewer (IGV). TSS1 and TSS2 denote known TSS (or proximal TSS) and novel TSS (or distal TSS), respectively. Green rectangles represent Smyd3 binding motifs reported by Hamamoto et al (Hamamoto et al., 2004a). Red arrow pairs denote primer pairs used for ChIP‐PCR in panel B. (b) H3K4me3 level and Smyd3 binding intensity at the promoter region of p21 in Ang II‐treated (Ang II) and control RAECs evaluated by ChIP‐PCR. mRNA (c) and protein level (d) of p21 in Ang II‐treated and control RAECs quantified by qRT‐PCR and Western blot, respectively. ^**^ *p* < 0.01 based on at least three different replicates, with representative blots image was shown. (e) Diagrammatic sketch of the two transcript variants derived from TSS1 and TSS2 with the same coding region (blue rectangle) but distinct 5´UTRs (thin orange and purple rectangle). (f) p21 protein level, (g) SA‐β‐Gal staining with the percentage of the SA‐β‐Gal positive cells, and (h) EdU incorporation with the quantification of percent EdU^+^ cells in p21 knockdown (siRNA) and control (si Scr) RAECs before and after Ang II treatment. All images shown are representative ones from at least three replicates, results shown as mean ± SEM, ^**^ *p* < 0.01, ^***^ *p* < 0.001, (*n* ≥ 3).

We next narrowed down the direct target genes of Smyd3 using the following three criteria: (a) increased H3K4me3 level in the promoter region; (b) upregulated mRNA level; (c) DNA‐binding motif of Smyd3 (5′‐CCCTCC‐3′ or 5′‐CCCCTC‐3′) exist near the promoter region of the target gene (Hamamoto et al., 2004a). With these three criteria, we finally screened out 57 genes (Figure S9), among of which is the well‐known senescence‐associated gene, *Cdkn1a* encoding p21 (Figure 4a). Interestingly, based on the RNA‐seq track, p21 seemed to have a novel transcriptional start site (TSS2, or the intronic TSS), wherein a slight elevation of H3K4me3 level was also seen in Ang II‐induced cells compared to control ones (Figure 4a). The increase of H3K4me3 levels near these two promoters in Ang II‐treated cells was further validated by ChIP‐PCR (Figure 4b), and H3K4me3 showed a more obvious increase around TSS1 than that of TSS2 (Figure 4b), in line with the ChIP‐seq visualization (Figure 4a). There are three and two Smyd3‐binding motifs near TSS1 and TSS2 of *Cdkn1a*, respectively (Figure 4a). The direct binding of Smyd3 to the two promoter regions of *Cdkn1a* was further confirmed by ChIP‐PCR (Figure 4b, the quantification was shown in Figure S10). In addition, the increased expression of p21 upon Ang II treatment was also confirmed at both mRNA (Figure 4c) and protein level (Figure 4d). Of note, the novel transcript variant (the one derived from TSS2) shared the same coding sequence (CDS) as the annotated one using TSS1 (leading to the same protein product), although they have distinct 5′ untranslated regions (5′ UTRs) (Figure 4e, Figure S11). These results above indicate that Smyd3 can promote p21 expression possibly via its H3K4 methylation‐dependent function.

To examine whether elevated p21 expression can lead to vascular senescence, we overexpressed p21 (p21‐OE) in RAEC cells (Figure S12A). p21‐OE RAECs showed typical senescence phonotypes of increased SA‐β‐Gal activity (Figure S12B) and decreased EdU incorporation (Figure S12C), which could all be reversed by knockdown of p21 (Figure 4f–h). Intriguingly, knockdown of p21 also reversed endothelial senescence markers p16 and COX‐2 induced by Smyd3 overexpression (Figure S13), suggesting that p21 plays an important role in mediating vascular senescence. Together, these data demonstrate that Smyd3 binds to the promoter regions of p21 gene, leading to elevated H3K4me3 occupancy and increased expression of p21, which in turn causes senescence‐related phenotypes.

### 2.5 Knockdown or inhibition of Smyd3 rescues senescence‐associated phenotypes in Ang II‐infused mice

**Figure 5:**
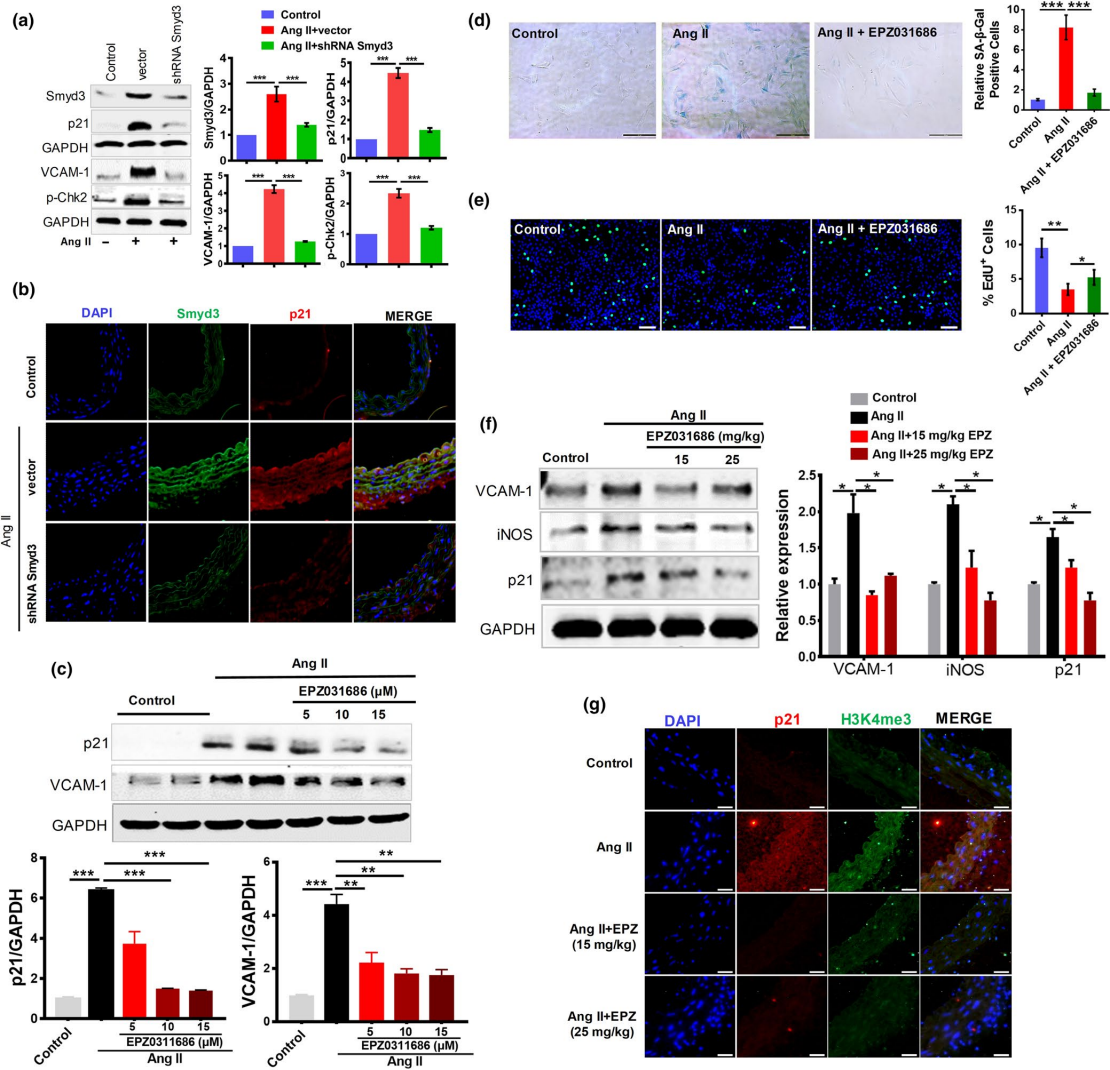
Smyd3 knockdown and Smyd3 specific inhibitor EPZ031686 rescue vascular aging in Ang II‐infusion mice. (a and b) Smyd3 knockdown prevents vascular aging in Ang II‐infusion mice. (a) The abundance of four proteins (Smyd3, p21, VCAM‐1 and p‐Chk2) was assayed by Western blot in Smyd3 knockdown (shRNA Smyd3) and control (vector) mice with or without Ang II infusion. GAPDH serves as internal control. *n* = 6/group, ^**^ *p* < 0.01; (b) Immunofluorescence staining of Smyd3/p21 in aortic great vessels of Smyd3 knockdown (shRNA Smyd3) mice and control (vector) mice with or without Ang II infusion. Visualization was realized with microscopes at 200× magnification. (c–e) EPZ031686 attenuates Ang II‐induced senescence of RAECs. Protein levels of p21, VCAM‐1, and GAPDH in RAECs treated with EPZ031686 at different concentration, evaluated by Western blot. The results (with the representative images shown) were based on at least three experiments, and the data are shown as the mean ± SEM, ^**^ *p* < 0.01 and ^***^ *p* < 0.001, respectively (c); SA‐β‐Gal staining with the percentage of the SA‐β‐Gal positive cells (d) and EdU incorporation assay with quantification of percent EdU^+^ cells (e) in RAECs treated with different combinations of Ang II and EPZ031686, shown as mean ± SEM, ^*^ *p* < 0.05, ^**^ *p* < 0.01, and ^***^ *p* < 0.001 (*n* ≥ 3). (f–g) EPZ031686 attenuates vascular aging in Ang II‐infusion mice. (f) Protein levels of p21, VCAM‐1, iNOS and GAPDH in aortic great vessels of mice treated with combinations of Ang II infusion and different dosages of EPZ031686 (15, 25 mg/kg/day), as evaluated by Western blot; *n* = 6/group, ^*^ *p* < 0.05; (g) Immunofluorescence double staining of H3K4me3 and p21 in aortic great vessels of mice treated with different combinations of Ang II and EPZ031686 (EPZ)

To explore whether the intervention of Smyd3 can be a potential strategy for preventing vascular aging, we performed lentivirus‐mediated knockdown of Smyd3 (Smyd3‐KD) in mice. Surprisingly, Smyd3 knockdown significantly alleviated the senescence‐related phenotypes in arteries of 4‐week Ang II‐infusion mice (Figure 5a). Furthermore, Smyd3‐KD resulted in decreased Smyd3 and p21 protein expression in the cross‐sectional area of blood vessels determined by immunofluorescence analysis (Figure 5b). Meanwhile, Smyd3‐KD showed decreased H3K4me3 expression in blood vessels (Figure S14). These results suggest that reduced expression of Smyd3 repressed p21 protein level and other vascular senescence markers in vivo.

More excitingly, EPZ031686 (the Smyd3 specific inhibitor) can alleviate Ang II‐induced senescence in RAECs, represented by the changes in multiple senescence markers including downregulated p21 protein level, reduced vascular aging marker VCAM‐1, decreased SA‐β‐Gal activity, and increased EdU incorporation (Figure 5c–e). Moreover, we also examined whether Smyd3 inhibition by EPZ031686 could reverse the expression of senescence markers in Ang II‐infusion mice. After a 4‐week treatment with EPZ031686, the senescence‐related phenotypes in arteries of Ang II‐infusion mice were significantly alleviated (Figure 5f). Furthermore, mice treated with EPZ031686 showed decreased H3K4me3 and p21 protein expression in the vascular wall (Figure 5g). All these data above suggested that Smyd3 inhibitor could reverse Ang II‐induced senescence phenotypes both in vitro and in vivo.

### 2.6 Smyd3 knockout (Smyd3^−/−^) mitigates senescence phenotypes in Ang II‐infusion mice

**Figure 6:**
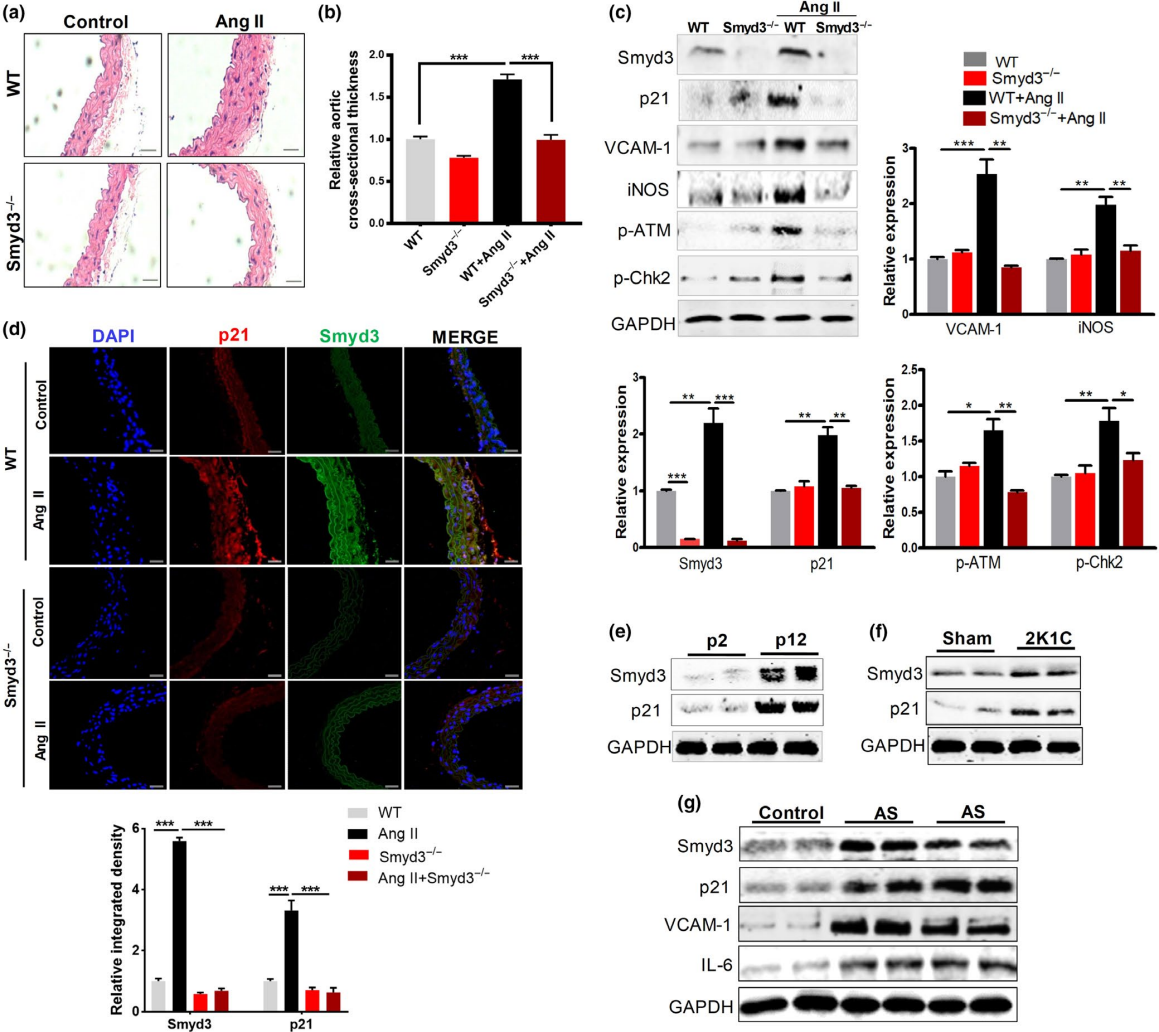
Smyd3 deficiency attenuates Ang II‐induced vascular aging and Smyd3 upregulation exists also in cellular replicated senescence models or aging‐related disease models. (a–d) Smyd3 deficiency attenuates Ang II‐induced vascular aging. (a) Representative hematoxylin and eosin (H&E) staining of thoracic aorta sections from wild‐type (WT) or Smyd3 knockout (Smyd3^−/−^) mice; (b) The relative thickness of cross‐sectional area was quantified. Data are presented as group mean ± SEM ^***^ *p* < 0.001 (*n* = 6/group); (c) protein expression of Smyd3, p21, VCAM‐1, iNOS, p‐ATM, and p‐Chk2 in aortic great vessels of Ang II‐infused WT and Smyd3^−/−^ mice (*n* = 6/group), as evaluated by Western blot. ^*^ *p* < 0.05, ^**^ *p* < 0.01, ^***^ *p* < 0.001; (d) Immunofluorescence double staining of Smyd3 and p21 in aortic great vessels of WT and Smyd3^−/−^ mice (*n* = 6/group), ^***^ *p* < 0.001. (e–g) Smyd3 upregulation also exists in both replicated senescence models and aging‐related disease models. (e) Protein level of Smyd3 and p21 in early passage (p2) and late passage (p12) of RAECs assayed by Western blot; (f) Protein level of Smyd3 and p21 in arteries of hypertensive rat model (2K1C) and control rat (Sham) assayed by Western blot; (g) Protein level of Smyd3, p21, VCAM‐1 and IL‐6 in thoracic aorta in healthy (control) and atherosclerotic (AS) patients evaluated by Western blot. Six samples from AS patients undergoing endarterectomy of elastic arteries were divided into 2 batches (3 samples each as experimental replicates), while 3 samples collected from organ donors act as the control group. GAPDH serves as the internal control for all Western blot

To further define the role of Smyd3 in Ang II‐induced senescence, we established Smyd3 knockout (KO) mice and treated them with Ang II for 28 days. Interestingly, Ang II‐induced increase in aortic cross‐sectional thickness was significantly reduced in Smyd3**‐**KO mice, as determined by H&E staining (Figure 6a,b). As expected, Smyd3‐depleted mice had significantly decreased p21 expression in the aorta of Ang II‐treated mice compared to WT mice (Figure 6c), suggesting Smyd3 depletion in vivo blocked the expression of p21. We next examined the aortic expression of various proinflammatory molecules, the result showed that Ang II treatment strikingly induced the expression of proinflammatory mediator, iNOS and VCAM‐1, in the aortas of WT mice, whereas Smyd3 depletion effectively blocked the Ang II‐induced expression of these molecules (Figure 6c). Likewise, Ang II markedly increased the DNA damage markers p‐ATM and p‐Chk2 in WT mice but not in Smyd3^−/−^ mice (Figure 6c). Of note, Smyd3 depletion also resulted in decreased p21 protein expression in the cross‐sectional area of blood vessels of Ang II‐infusion mice (Figure 6d). Moreover, proportion of positive stain for leukocytes (CD45^+^), the common leukocyte antigen marker (Hazan et al., 2010), significantly increased within the endothelial zone in the vascular wall of Ang II‐infusion mice compared to the Ang II‐free controls (Figure S15). These results above suggest that Smyd3 depletion could efficiently prevent Ang II‐induced senescence in mice. Furthermore, systolic blood pressure (SBP) in conscious mice was measured by the tail‐cuff method in both WT and Smyd3^−/−^ mice treated with 4‐week Ang II infusion. The results showed that Ang II‐infused Smyd3^−/−^ mice exhibited much decreased SBP compared to Ang II‐infused WT mice (Figure S16A), indicating Smyd3 is directly responsible for the Ang II‐induced blood pressure increasing in vivo. In addition, by measuring the acetylcholine (Ach)‐induced vasorelaxation in descending aortic arteries using an organ chamber, the blood vessels reactivity was also examined in both Smyd3^−/−^ and WT mice with 4 week Ang II‐infusion. We found that the Ach‐induced endothelial‐dependent relaxation (EDR) in the aortic artery was improved in Ang II‐infused Smyd3^−/−^ mice compared to Ang II‐infused WT mice (Figure S16B). These results suggest that Smyd3 mediates Ang II‐induced blood pressure increase and endothelial dysfunction.

### 2.7 Smyd3‐mediated p21 upregulation also exists in other aging‐related disease models

We next ask whether such Smyd3‐mediated p21 upregulation also exists in other cellular senescence models and vascular aging‐related disease models. In the replicative senescence model of RAECs, we found that Smyd3 and p21 expression significantly increased at a later passage compared to the earlier one (Figure 6e). Since senescence has been detected in cells at sites of arteries prone to atherosclerosis or chronic arterial hypertension in human and experimental animal (Wang, Kim, Monticone, & Lakatta, 2015), we are curious whether Smyd3 and p21 also showed upregulated expression in related models. First, two‐kidney, one‐clip renovascular hypertensive rats (2K1C) showed higher expression of Smyd3 and p21 at protein level in diseased arteries compared to control rats (Figure 6f). Second, human atherosclerotic samples demonstrated higher expression of Smyd3, p21, VCAM‐1, and IL‐6 compared to healthy thoracic aorta (Figure 6g). Lastly, relatively aged rats (10 months) showed upregulated Smyd3 expression, along with the elevated protein expression of p21, p16, COX‐2, and IL‐6 compared to younger rats (3 months), suggesting that Smyd3 may also contribute to vascular aging at individual level (Figure S17). All these additional results above extended our findings for the role of Smyd3 and p21 in vascular aging and related diseases, combined to indicate the regulatory role of Smyd3‐p21 axis in hypertension and atherosclerosis, implying their biomedical importance in therapy of vascular diseases.

## 3 DISCUSSION

Aging, a known cardiovascular risk factor, is associated with progressive structural and functional changes in vasculature. The effect of aging on cardiovascular health is in part because that aging perturbs a number of metabolic and hemodynamic environment in the cardiovascular system, especially the vascular endothelium (Lakatta & Levy, 2003b). Notably, the endothelium (the innermost layer of the vessel wall) becomes senescent and progressively dysfunctional with aging. Of note, endothelial dysfunction is one of the earliest indicators of cardiovascular disease, and endothelium has emerged as one of the most important targets for the prevention and treatment of multiple cardiovascular diseases (such as hypertension, diabetes, atherosclerosis, and insulin resistance syndrome) (Lakatta & Levy, 2003a; Pinto, 2007). Thus, it is of great importance to seek potential target affecting endothelial senescence. Age‐dependent changes in epigenetic regulation have been observed in multiple senescence and aging models (Zhang, Song, Qu, & Liu, 2018), suggesting the important role of epigenetic factors in senescence and aging. Different epigenetic modifications can interact with each other to regulate chromatin structure, gene expression, telomere function, DNA damage, and oxidative stress, all of which can contribute to cell senescence, even tissue aging. However, whether epigenetic factors play a role in vascular aging and whether such factors can be manipulated to intervene cellular senescence are largely unknown. Our previous studies showed that cardiac angiotensin‐converting enzyme played critical roles in various cardiovascular diseases (Wang, Khazan, & Lakatta, 2010), indicating the biomedical importance of Ang II‐induced endothelial senescence. Thus, in the present study, we have focused on screening for key histone modifiers by using Ang II‐induced RAEC and other vascular senescence models and found that the epigenetic writer, Smyd3, showed significantly increased expression during Ang II‐induced senescence. We further demonstrated that Smyd3 played a novel role in promoting vascular senescence by transcriptionally activating p21 through binding to its promoter and specifically promoted endothelial cell senescence (Figure 4). Interestingly, such mechanism exists in multiple senescence/aging models, including Ang II‐induced rat cell model, replicative cell model, Ang II‐infused mouse model, hypertension rat model, and human atherosclerotic samples. Moreover, intervention of Smyd3 exhibited the capacity of preventing vascular cell senescence, indicating its potential to treat vascular aging‐related diseases. Our work thus reveals a novel epigenetic mechanism in regulating vascular senescence, which may also have biomedical implications in treating aging‐related vascular diseases.

To explore whether Smyd3‐mediated regulation of p21 gene (*Cdkn1a*) is evolutionary conserved, we compared the gene structure and H3K4me3 binding site of *Cdkn1a* in mice, rat, and human. Interestingly, the proximal TSS of *Cdkn1a* was supported by annotation of in GenBank or RefSeq database for these three species (Figure S18). According to RefSeq annotation in human, *Cdkn1a* have two (proximal and distal) TSSs, which were both occupied by H3K4me3 marks nearby in multiple cell lines (Figure S19A), indicating they are both genuine TSSs. In addition, each of the two TSSs has two upstream Smyd3 binding motifs (Figure S19B). Interestingly, these stays true for mice *Cdkn1a* (Figure S20A,B). Of note, according to RefSeq gene annotation, the two transcript variants of *Cdkn1a* caused by alternative promoter usage share the same CDS but different 5′ UTRs in both human and mouse. These data, along with Smyd3‐mediated upregulation of p21 in mouse model, rat cell and hypertensive model, and human atherosclerotic sample, combined to indicate that the regulatory mechanism found here in rat might be evolutionarily conserved and it possibly can be extended to human physiological/pathological processes related to vascular senescence.

Previous studies indicate that Smyd3 can regulate cancer‐associated phenotypes through its enzymatic activities including methylation of histones (such as H3K4, H4K5, and H2A.Z) and non‐histone proteins (such as VEGFR and MAP3K2) (Kunizaki et al., 2007). H3K4me3 showed increased total abundance and higher binding intensity at the promoter regions of target genes in Ang II‐induced senescent endothelial cells, wherein, Smyd3 also showed increased protein level. Given the H3K4 methyltransferase role of Smyd3, we hypothesize that Smyd3 could be involved in regulating endothelial senescence through modifying H3K4 at certain target genes. Supporting this hypothesis, genes with higher H3K4me3 level and increased mRNA expression upon Ang II induction were enriched in senescence‐associated pathways. In addition, p21 was implicated in cell cycle arrest and cellular senescence (Cheung et al., 2012) and we discovered that p21 can be directly regulated by Smyd3 and contribute to senescence. Of note, though all the evidences in our present study demonstrated that Smyd3 may contribute to p21 transcription through the H3K4 methylation function in Ang II‐induced RAECs senescence. Actually, we cannot rule out the possibility that methylation of other histones (H4K5 and H2A.Z) or non‐histone proteins may also contribute to Ang II‐induced senescence. A comprehensive investigation of related chemical modifications and their possible interactions are definitely required to fully understand the mechanism by which Smyd3 regulates senescence.

Besides, the direct evidence of Smyd3 mediated H3K4me3 methylation in the present data is absent. A Smyd3 methylation mutant should be conducted to claim that Smyd3 is the main H3K4me methylase in Ang II‐induced senescence, which should be further explored in future studies.

Though we discovered that Smyd3 affected senescence through regulating p21 expression, we admitted that p21 might not be the only contributor in mediating Smyd3 induced senescence. We did identify 57 possible direct targets that can be regulated by Smyd3‐dependent H3K4me3 modification. In this study, the candidate gene *Cdkn1a* (encodes p21) was selected for intensive investigation. Knockdown of p21 rescued Ang II‐induced senescence‐associated phenotypes, supporting that Smyd3‐p21 axis makes a considerable contribution to senescence. Other target genes also deserved functional and mechanism investigation to screen for other possible contributors.

Multiple upstream factors can regulate p21 expression (Gartel et al., 2001; Munoz‐Espin & Serrano, 2014). Among them, p53 is the most famous one. To examine whether Smyd3‐mediated upregulation of p21 is p53 dependent in Ang II‐induced endothelial cell senescence, we quantified the protein level of both p21 and p53 in Smyd3 overexpressed RAEC cells. Interestingly, upregulation of Smyd3 did not increase p53 level but promoted p21 level. Further, knockdown of p53 did not block the upregulation of p21 induced by Smyd3 overexpression (Figure S21). These data indicate that Smyd3‐driven p21 upregulation is independent of p53 at least in Ang II‐induced endothelial senescence model.

## 4 CONCLUSIONS

We discovered Smyd3 as a novel epigenetic regulator for endothelial senescence. Upregulation of Smyd3 promoted endothelial senescence while downregulating or inhibiting Smyd3 attenuated the development of senescence‐associated phenotypes both in vitro and in vivo. Smyd3‐mediated transcriptional upregulation of p21 independent of p53 makes up at least one of the mechanisms how Smyd3 is involved in regulating endothelial senescence. Targeting Smyd3 may represent a novel therapeutic approach for vascular aging‐associated diseases.

## 5 MATERIALS AND METHODS

All supporting information can be found online in the Supporting Information files, wherein, a detailed description of methods and materials is also provided.

## CONFLICT OF INTEREST

The authors have declared no conflict of interests.

## AUTHOR CONTRIBUTIONS

D.Y. and X.H.L. designed experiments. L.F.Q. provided the clinical tissues of the human patients. X.L.T. and W.J.H. gave valuable suggestions on the research. D.Y., G.W., F.L., H.B.N., S.X.W., P.L., Y.Q. and Y.W. performed experiments. D.Y., G.W., T.N. and X.H.L. analyzed data. D.Y., G.W., T.N., X.H.L. and Y.Z.Z. wrote the manuscript.

## Supporting information

Supplementary Material

## Data Availability

The data that supports the findings of this study are available in the supplementary material.
