# Supplementary material for: Histone methyltransferase Smyd3 is a new regulator for vascular senescence
Source: Aging Cell. 2020 Aug 11;19(9):e13212. doi: 10.1111/acel.13212 (PMC7511874; doi:10.1111/acel.13212)
Supplement: Supplementary file 1 — Supplementary Material [file ACEL-19-e13212-s001.docx]

**SUPPORTING INFORMATION**

**Supplemental Methods**

**Reagents**

Reagent sources were as follows: angiotension (Ang II) was from Meilunbio (Meilun Biotechnology, Dalian, China). EPZ031686 (Smyd3 inhibitor) was purchased from MedChem Express (MCE, USA). DAF-FM DA Kit (S0019) was purchased from Beyotime (Shanghai, China) and Dihydroethidium (DHE) Kit (50102ES02) was purchased from Yeasen (Shanghai, China). Antibodies were obtained from the following commercial sources: Smyd3 (GTX121945) were purchased from GeneTex (Alton Pkwy Irvine, CA, USA); p21 (ab109199), H3K4me3 (ab8580), H3K4me1 (ab8895), H3K36me2 (ab9049) and H3K27ac (ab4729) were purchased from Abcam (Cambridge, MA, USA); GAPDH (60004-1-Ig), p16 (10883-1-AP), VCAM-1(11444-1-AP) and iNOS (18985-1-AP) was purchased from Proteintech (Rosemont, IL, USA); COX-2 (A1253), IL-6 (A2447) was purchased from ABclonal (Wuhan, China); H3K27me3 (9733s), Smad3 (5678), p-Smad3 (8828), γ-H2AX (5438) and p53 (2524S) were purchased from Cell Signaling Biotechnology (Danvers, MA, USA); p-ATM (#AB11122) and p-Chk2 (#AB12191) were purchased from Absci (MD, USA); eNOS (GB11086) and CD45 (GB11066) were purchased from Servicebio (Servicebio Technology, Wuhan, China); H3K9me3 (07-523) and p-eNOS (07-428) were purchased from Merck (Merck, USA).

**Ethics statement**

Male C57BL/6 mice (22-25 g) and Sprague-Dawley rats were purchased from SHANGHAI SLAC LABORATORY ANIMAL CO. LTD (Shanghai, China). All animals were housed under conventional conditions in the animal care facilities and received humane care in compliance with the Principles of Laboratory Animal Care formulated by the National Society for Medical Research and the Guide for the Care and Use of Laboratory Animals. The experimental protocol conformed to the Animal Welfare Act Guide for Use and Care of Laboratory Animals, and was approved by Institutional Animal Care and Use Committee (IACUC), School of Pharmacy, Fudan University, China. Plaque specimens of 6 patients undergoing endarterectomy of elastic arteries were analyzed in our present study. Samples in control group were collected from organ donors. The study was approved by the ethics committee of Changzheng hospital, The Second Military Medical University, and all patients gave informed consent.

**Generation of Smyd3 knockout mice**

To generate mice with a conventional knockout allele for Smyd3 (Smyd3-KO), a Streptococcus pyogenes Cas9 (SpCas9) target site in the conserved exon 1 was selected. The mRNA encoding the Cas9 nuclease and the short guide RNA (sgRNA) targeting Smyd3 were injected into zygotes from a C57BL/6 background. The genotypes of the generated mice were identified and confirmed by PCR assay using total DNA isolated from tails. The primers Smyd3-P1 and Smyd3-P2 were used to identify the deletion product. The primers used for identification are as follows: M-SMYD3-chk-F1: 5’- GCGCAGTCAGAAAACACGAG-3’ and M-SMYD3-chk-R1: 5’- ATTATCTGCCCGACGCTTCC -3’. Founder animals were identified by southern blotting and crossed with F1 mice to generate lines. The resulting Smyd3-KO mice were devoid of Smyd3 mRNA and protein in all tissues, including arteries.

**Animal treatment**

Minipump (Model 2004, ALZA Scientific Products, Mountain View, CA, USA) were implanted subcutaneously in male mice to deliver Ang II (1.5 mg/kg/day) or vehicle (sodium chloride) for 4 weeks. Six male mice was given a daily by intragastric administration with EPZ031686 (15 and 25 mg/kg of body weight) until the end of the experiment on day 28 after infusion of Ang II. Six mice were intravenously injected via the tail vein 4 time with lentiviral vector expressing shRNA Smyd3 (KD Smyd3) or shRNA scramble for every 7 days (began at -1 day) and then infused with Ang II or vehicle, each injection contains 0.15 mL of the concentrated viral suspension with a titer of 1×10^8^ IU/mL.

C57BL/6 mice were sacrificed by cervical dislocation and the thoracic aorta of each mouse was cut into two sections. One section was stored at -80°C for protein extraction. The other section was fixed with 4% paraformaldehyde overnight, and was then embedded in paraffin for immunofluorescence staining. Paraffin-embedded sections (8 μm thick) were cut every 200 μm length of thoracic aortas from the proximal thoracic aortas specimens.

The two-kidney, one-clip (2K1C) hypertension was induced as described previously ([Cervenka, Wang, Mitchell, & Navar, 1999](#_ENREF_2)). Briefly, male SD rats (initial body weight 200-250 g) were anesthetized with pentobarbital sodium (40 mg/kg, *i.p.*). The right renal artery was isolated through a flank incision and a clip (0.2 mm internal diameter) was placed on the renal artery. Sham-operated rats underwent the same surgical procedure except placement of the arterial clip. After 28 days, all the animals were sacrificed and arteries were collected from the nonclipped kidney of 2K1C rats and from the left kidney of sham-operated rats. The average systolic blood pressure of our 2K1C rats was 190 ± 3 mmHg and of sham-operated rats 113 ± 2 mm Hg.

**Blood pressure and endothelium-dependent vasorelaxation measurements**

The systolic blood pressure (SBP) in conscious mice was measured by the tail-cuff method after 28 days of Ang II infusion in both wild-type (WT) or Smyd3 knockout (Smyd3^-/-^) mice using a non-invasive computerized tail-cuff system (BP-2000, Visitech Systems, Apex, NC).

The blood vessels reactivity after 4 weeks of Ang II-infusion in both WT or Smyd3^-/-^ mice by measuring acetylcholine (Ach)-induced vasorelaxation in descending aortic arteries were performed with the help of Dr. Shuangxi Wang’s and Dr. Peng Li’s group. Briefly, the representative aortic rings were cleaned and cut into 3-mm length rings. Then the aortic rings were mounted in an organ chamber with Kreb buffer in a myograph system (Danish Myo Technology A/S, Aarhus, Denmark). Contractile response was evoked by treatment with phenylephrine (1 μmol/L). At the plateau of contraction, accumulative Ach was added to establish an endothelium dependent vasorelaxation (EDR) curve. The relaxation was calculated as a ratio of ACh-induced vasodilation to phenylephrine-elicited vasoconstriction, and the ratio at 1 was set as 100% of relaxation.

**Culture of rat primary aorta endothelial cells (RAECs)**

Rat primary endothelial cells (RAECs) were isolated from male rats according to the method described previously ([Yang et al., 2018](#_ENREF_12)). The purity of these cultures was greater than 95% RAECs as identified by both positive staining for CD31 and morphologically based on their classical ‘hill and valley’ appearance. Cells that experienced 3-5 passages were used for Ang II-induced senescence experiments performed in this manuscript.

For the replicative senescence, the cells then underwent serial passaging to obtain senescence. The senescent status was verified by staining for SA-β-Gal. 90% percent of the cells at 12 passages stained positive for SA-β-Gal.

**SA-****β-Gal staining**

The expression of senescence-associated β-Gal in cells was determined by SA-β-Gal staining as described ([Itahana, Campisi, & Dimri, 2007](#_ENREF_4)).

**Western blotting**

Samples were prepared from cells and tissues lysed with RIPA buffer (Pierce, Rockford, IL, USA) containing protease and phosphatase inhibitor cocktail (Sigma, St Louis, USA). Whole lysates samples were separated by SDS-PAGE and blotted to nitrocellulose membrane. Protein bands were detected with fluorophore-conjugated secondary antibodies, and detection and analysis were performed with the Odyssey imaging system (LI-COR).

**Immunofluorescence staining**

The arterial sections were blocked in PBS with 10% goat serum for 30 min and incubated overnight with primary antibodies at 4°C. Cells were seeded on glass coverslips placed in 24-well plates. Cells were fixed with 4% paraformaldehyde for 15 min, followed by permeabilization with 0.25% Triton X-100 in PBS 10 min. Next, the slides were blocked in PBS with 10% goat serum for 30 min, and incubated overnight with primary antibodies at 4°C. Appropriate secondary antibodies were added and incubated with cells for 1.5 h at room temperature. The nuclei were stained with DAPI. The images were captured by using a fluorescence microscope (Axio Scope.A1, Carl Zeiss Imaging Systems).

For cellular NO detection, RAECs were incubated with medium containing 5 µM DAF-FM DA (Beyotime, S0019) at 37°C for 20 min. For cellular ROS detection, 10 µM Dihydroethidium (DHE) (Yeasen, 50102ES02) was added to incubate RAECs for 60 min at 37°C. After staining, RAECs were washed 3 times with PBS and the fluorescent intensity was detected by using a fluorescence microscope (Axio Scope.A1, Carl Zeiss Imaging Systems).

**Immunohistochemistry staining**

Infiltration of leukocytes around and within the vessel walls of Ang II-infusion mice was determined by immunohistochemistry stained with CD45 antibody. Briefly, Paraffin-embedded arterial sections were deparaffinized and retrieved the antigens by incubating arterial sections with citrate buffer at 95°C for 10 min. After blocking in 5% goat serum in PBS for 30 min, the CD45 antibody was used to incubate arterial sections at 4°C overnight. Then the arterial sections were incubated with appropriate secondary antibody for 1.5 h, followed by visualization with 3, 3-diaminobenidine (DAB). Image analysis was used to quantify proportion of CD45^+^ (leukocytes) staining surrounding vessels by a microscope.

**Plasma construction****, lentivirus generation and infection**

The Smyd3 and p21 cDNA expression plasmid was constructed using pcDNA3.1 backbone. Both plasmids were digested with EcoRI and BamHI (TaKaRa, China), and coding sequence (CDS) region of Smyd3 or p21 (GenBank: NM_001025762.1 and NM_080782.3) was subcloned into pcDNA3.1 vector to generate the recombinant vector pcDNA3.1- Smyd3 and p21. The Smyd3 and p21 CDS fragment was amplified from full-length cDNA.

To obtain the lentivirus, the recombinant plasmid, and packaging vector psPAX2 and Pmd2.G were co-transfected into 293T cells using transfection reagent lipofectamine 2000 (Invitrogen, USA). After incubation for 48 h, the lentivirus in the culture medium was collected by filtration with 0.45 μm filters. Lentivirus encoding Smyd3, or p21 or control vectors in the presence of 8 μg/ml polybrene (Sigma) was added to RAECs culture media for 24 h. This medium was then removed and the cells were allowed to incubate for additional 48 h under normal culture conditions.

**Small interfering RNA (siRNA) transfection**

Smyd3, p21, smad3, and p53 siRNA, and control siRNA were produced by GenePharma (Shanghai, China). To introduce siRNA into RAECs, the cells were plated on 6-well plates at 30% to 50% confluence before transfection. Corresponding siRNA (15 nM), Lipofectamine RNAiMAX and Opti-MEM were mixed and incubated at room temperature for 5 min. Then the siRNA-lipofectamine RNAiMAX complexes were added to cells and incubated for 24 h, then the medium was replaced with fresh serum DMEM medium. Experiments were performed 72 h after transfection.

**Proliferation analysis on RAECs**

To assess cell proliferation, immunofluorescence assay was also used for the detection of 5-Ethynyl-2’-deoxyuridine (EdU) incorporated into cellular DNA (with EdU Labeling and Detection Kit, KeyGEN BioTECH, Jiangsu, China). The immunofluorescence detection was performed according to the manufacturer's instructions. Total cellular nuclei were stained with DAPI. Zeiss inverted fluorescent microscope was used to detect immunofluorescence EdU positive cells.

**Quantitative real-time reverse transcription polymerase chain reaction (qRT-PCR) analysis**

Total RNA was extracted from RAECs with TRIzol Reagent (TaKaRa Biotechnology, Dalian, China) following the manufacturer’s instructions. Total RNA (2 μg) of each sample was reversely transcribed into cDNA and amplified using a PrimeScript 1st Strand cDNA Synthesis Kit (Takara). Expression levels of genes encoding proinflammatory mediators were quantified by qRT-PCR performed on an iCycler iQ system (Bio-Rad, Hercules, CA, USA). See Table S2 for the primer sequences.

**RNA-seq and ChIP-seq library construction**

PolyA+ RNA was enriched by oligo(dT)25 Dynabeads (Invitrogen) from total RNA extracted as above. The dUTP-based strand-specific RNA-seq libraries for control and Ang II induced RAEC cells were constructed according to the established protocol ([Parkhomchuk et al., 2009](#_ENREF_8)). ChIP-seq libraries were constructed according to the protocol established previously ([Barski et al., 2007](#_ENREF_1); [Wang et al., 2008](#_ENREF_11)), and H3K4me3 antibody (ab8580, abcam) was used during the immunoprecipitation step. After quality inspection, both RNA-seq and ChIP-seq libraries were sequenced on Illumina HiSeq platform, and paired-end reads of 150nt length were obtained.

**ChIP-PCR**

2.5-3 μg of anti-H3K4me3 (ab8580, abcam) and anti-Smyd3 (GTX121945, GeneTex) were diluted in BSA-PBS and then added to the Protein G Magnetic beads(#161-4023, Bio-RAD), then the complex were incubated overnight in rotation at 4◦C. Approximately 1.0 × 10^7^ RAECs were crosslinked with 1% paraformaldehyde (Sigma-Aldrich) for 15 min at room temperature. After quenching the paraformaldehyde reaction with 0.125 M glycine (Sigma-Aldrich) for 5 min, cells were harvested. Cells were washed twice with PBS and then lysed with lysis buffer I (NaCl 15 mM, KCl 60 mM, MgCl_2_ 5 mM, EGTA 0.1 mM, Sucrose 0.3 M, Tris-HCl-ph7.5 15 mM, 1×Protease inhibitor), lysis buffer II (NaCl 15 mM, KCl 60 mM, MgCl_2_ 5 mM, EGTA 0.1 mM, Sucrose 0.3 M, Tris-HCl-ph7.5 15 mM, 1×Protease inhibitor, 0.4% NP-40) and lysis buffer III (NaCl 15 mM, KCl 60 mM, MgCl_2_ 5 mM, EGTA 0.1 mM, Sucrose 1.2 M, Tris-HCl-ph7.5 15 mM, 1×Protease inhibitor) on ice and the chromatin was sonicated for 15 min in 12-s internals (12 s on and 12 s off) to shear chromatin into 300- to 500-base pair lengths. Ten percent of sample was then kept out as input, and the remaining sample was processed for the chromatin immunoprecipitation assays. Fragmented chromatin was subjected to immunoprecipitation with the antibody-Protein G Magnetic beads complexes and incubated overnight in rotation at 4◦C. Then the Protein-DNA crosslinks were reversed at 65◦C overnight. The immunoprecipitated DNA was purified and quantified by PCR amplification. Taq DNA Polymerase (Yeasen) was used for the PCR step, and PCR primers for selected genes were listed in Table S2. Thermal cycling was carried out as follows: 94°C for 30 s; 30 ~ 40 cycles of 94°C for 30 s, 52~58°C for 30 s and 72°C for 30 s; 72°C for 10 min; hold at 4°C. PCR product was visualized in a 2% agarose gel stained by EB dye. Values were normalized to input DNA and measurements were performed in technical triplicates.

**RNA-seq and ChIP-seq analysis**

The quality of the raw reads (of 150nt length) were first evaluated through FastQC (http://www.bioinformatics.babraham.ac.uk/projects/fastqc), and then the 3’ end 60nt were trimmed off to remove the nucleotide of low sequencing quality and adaptor sequence originated from the running off of relatively short inserted fragments. For RNA-seq data, the clean reads were first aligned to rat Unigene with Bowtie (v1.1.1) ([Langmead, Trapnell, Pop, & Salzberg, 2009](#_ENREF_5)) to evaluate the strand specificity and determine the average insert size and standard deviation required as a parameter by TopHat (-r and --mate-std-dev) ([Trapnell, Pachter, & Salzberg, 2009](#_ENREF_9)), then the reads of each library were aligned to the rat genome (Rnor6.0) using TopHat2 (v2.0.13). Cuffdiff, a sub-tool of Cuflinks (v2.2.1) ([Trapnell et al., 2010](#_ENREF_10)) was used to quantify and compare gene expression level (measured by FPKM, Fragments Per kilobase per Million reads). The differentially expressed genes (DEGs) was defined with the criteria that FPKM >1 in either the control or Ang-II treated sample, the fold change > 1.5 and the p < 0.05. The function classification and enrichment evaluation were performed on DAVID ([Huang da, Sherman, & Lempicki, 2009](#_ENREF_3)).

For the ChIP-seq data, the clean reads were aligned to rat genome with BWA (v0.7.10) ([Li & Durbin, 2009](#_ENREF_6)), the unique alignments were obtained through SAMtools (v0.1.19) ([Li et al., 2009](#_ENREF_7)) and custom scripts. To evaluate the reads distribution around transcription start sites (TSS), upstream and downstream 2 kb around the annotated TSS were extracted, split into 200 bins, and assigned with the unique alignments, then the aggregation plot was drawn to get an overall view of the difference of H3K4me3 distribution between the control and Ang-II treated samples. MACS2 (v2.1.1) ([Zhang et al., 2008](#_ENREF_13)) was used to identify the potential H3K4me3 peaks, and bdgdiff (a sub-tool of MACS2) was used to find the significantly different H3K4me3 distribution between the control and Ang-II treated samples. Regions of 1 kb upstream and 500 bp downstream TSS were defined as the promoter region and used to search for the potential binding site of Smyd3.

**Statistical analysis**

Experimental results were expressed as means ± S.E.M. Differences of means were analyzed by using one-way ANOVA with the Turkey-Kramer post hoc test for multiple group comparison, and unpaired Students *t*-test for two group comparison. The probability value *p* < 0.05 was used as the significance cutoff.

**Supplemental Figures:**


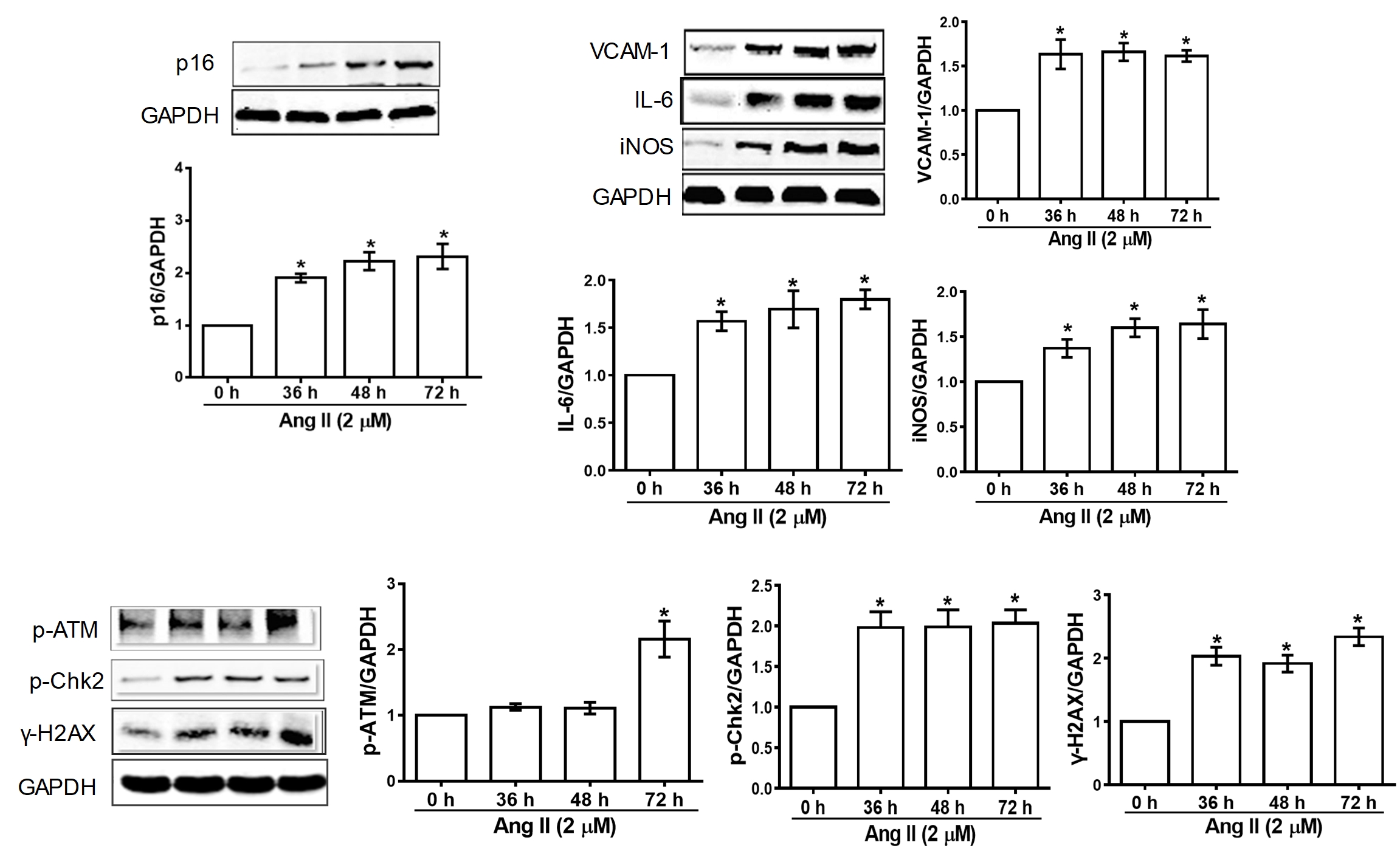


**Supplemental Figure 1.** Increased p16, VCAM-1, IL-6, iNOS, p-ATM, p-Chk2 and γ-H2AX expression by Western blot in 2 μM Ang II-induced RAECs for indicated time. Protein quantitative analysis was shown for each Western blot figure. GAPDH serves as the internal control. ^*^*p* < 0.05 based on t-test by comparing with untreated cells.

**
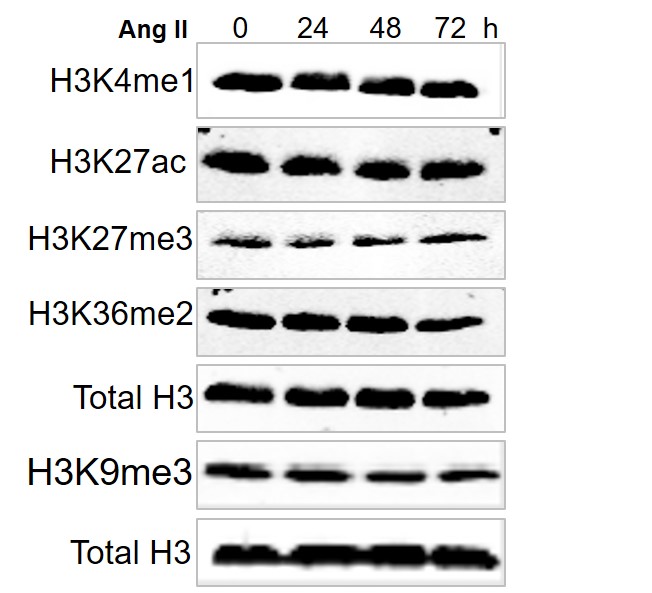
**

**Supplemental Figure 2.** Histone modifications including H3K4me1, H3K27me3, H3K27ac, H3K36me2 and H3K9me3 did not show significant increase in Ang II- treated RAECs. Total H3 serves as the internal control.


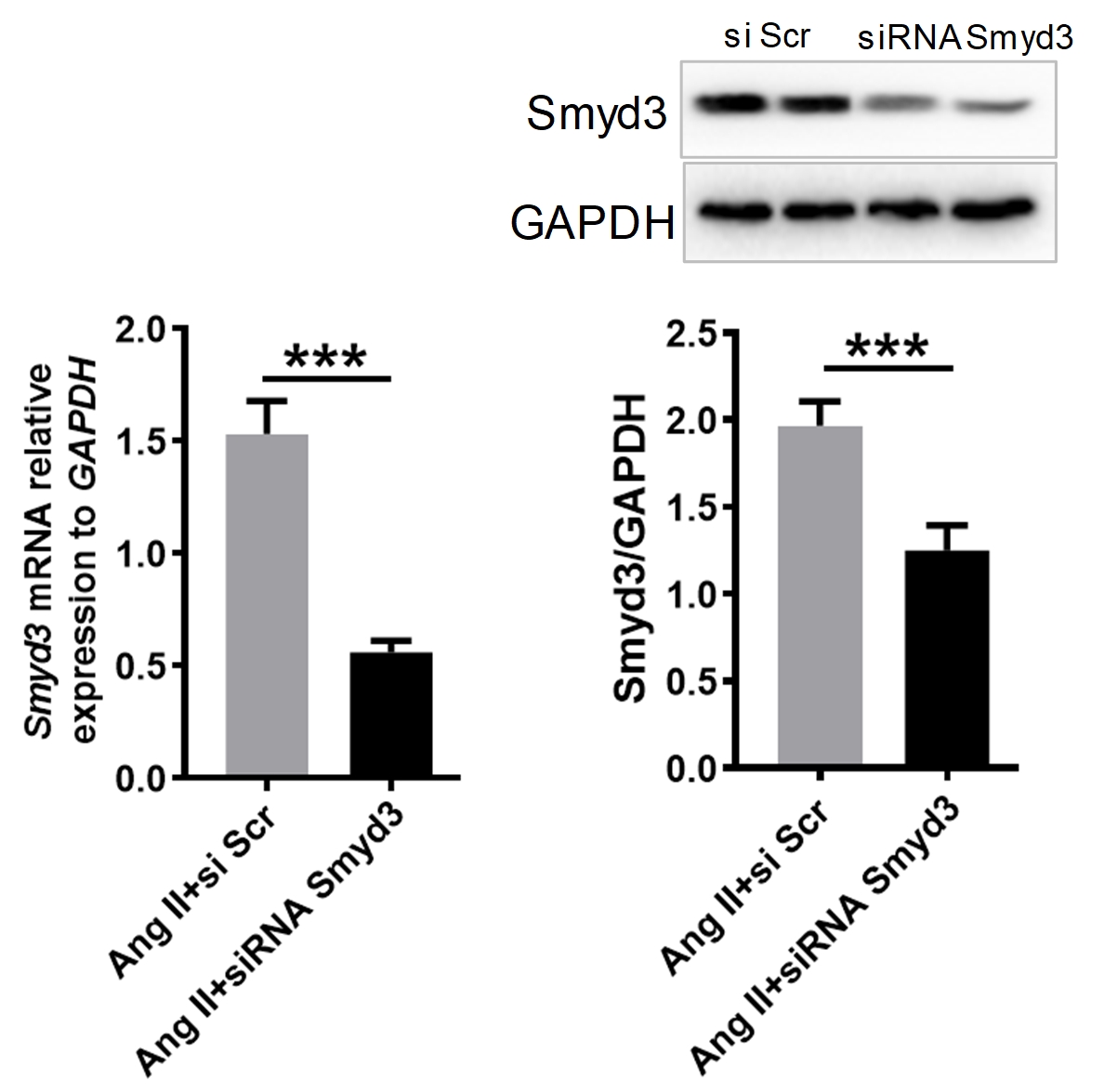


**Supplemental Figure 3. Evaluation of Smyd3 expression in Smyd3-KD cells treated with Ang II at both mRNA and protein level.** (A) RAEC cells were transfected with scramble or Smyd3 siRNA before and after Ang II induction. Smyd3 mRNA level was measured by Real-time PCR. (B) Smyd3 protein levels was detected by Western Blot. GAPDH serves as the internal control, ^***^*p* <0.001, all data were shown from at least three different replicates


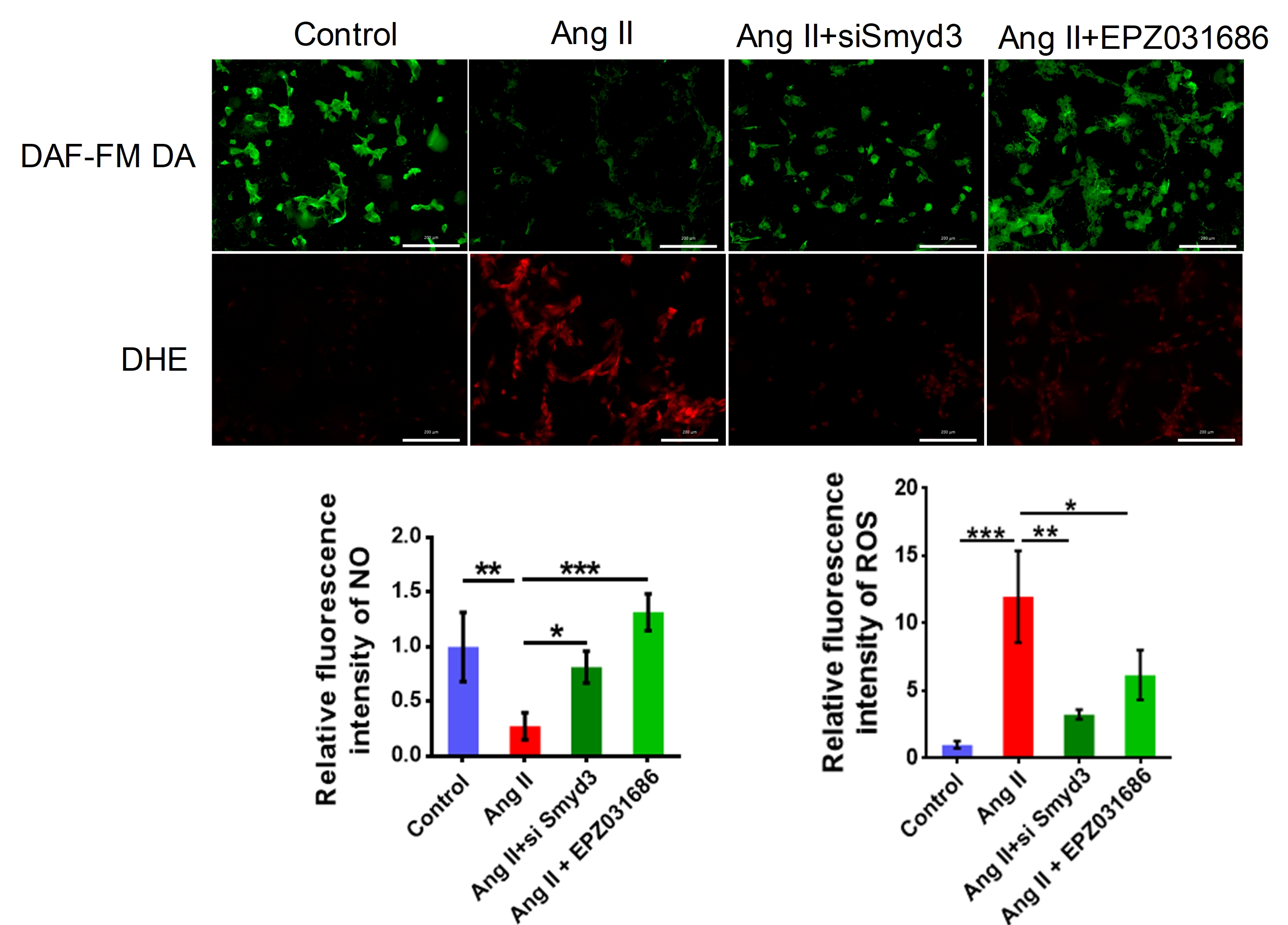


**Supplemental Figure 4.** Inhibition or knockdown of Smyd3 rescued Ang II induced-NO reduction and ROS production in RAECs and the quantification of relative fluorescence intensity of NO or ROS were shown below. RAECs were pretreated with Smyd3 siRNA or EPZ031686 (40 μM) and then stimulated by Ang II (2 μM) for 48 h. The production of NO and ROS was detected using DAF-FM DA Kit and Dihydroethidium (DHE) Kit, respectively. Results shown as mean ± S.E.M., ^*^*p* < 0.05, ^**^*p* < 0.01, ^***^*p* < 0.001, (n ≥ 3).


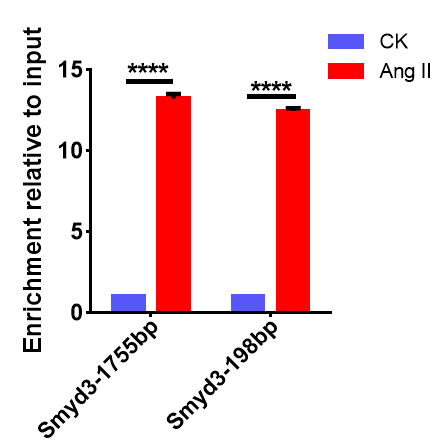


**Supplemental Figure 5.** The quantification of ChIP-PCR in Figure 3B. ^****^*p* <0.0001, data were shown from at least three different replicates.


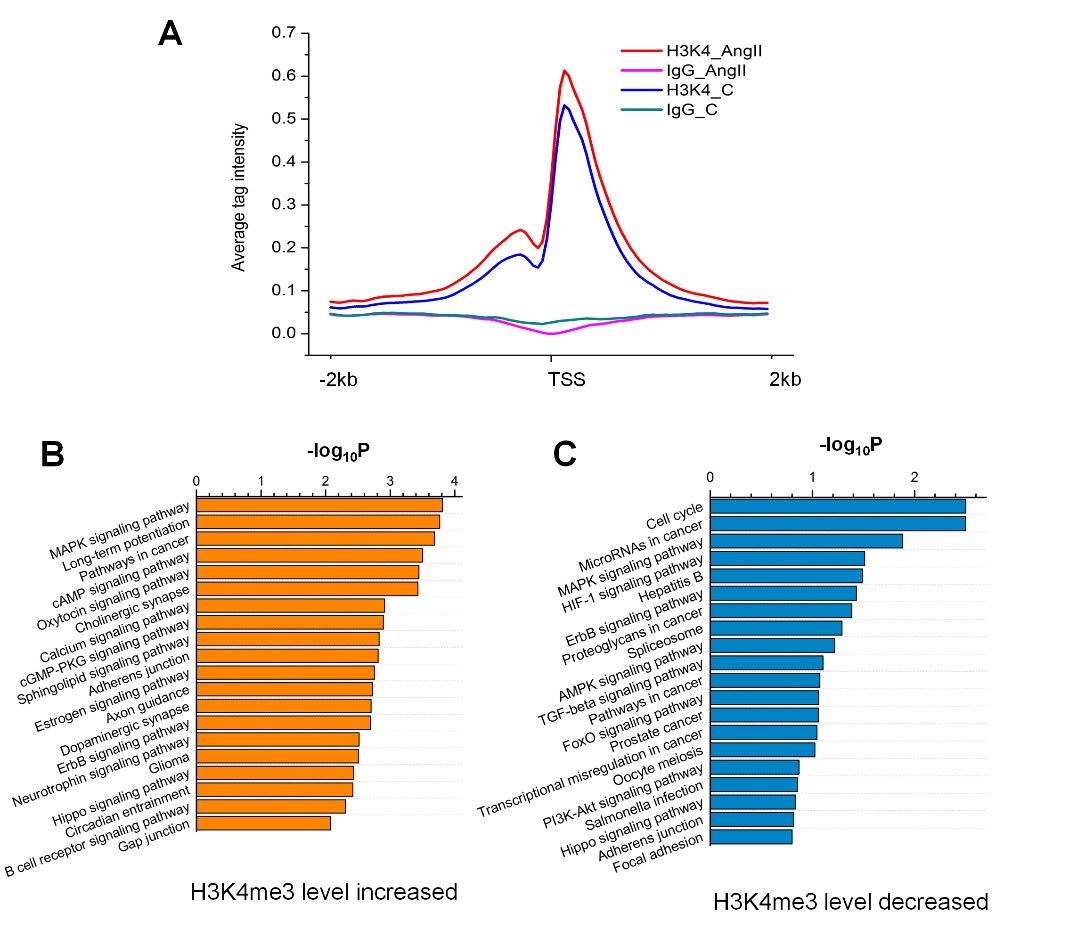


**Supplemental Figure 6.** Increased H3K4me3 level upon Ang II induction in RAECs and pathway enrichment analysis for genes with changed H3K4me3 accumulation. (A) Aggregation plot of H3K4me3 around TSS before and after Ang II treatment. IgG serves as a negative ChIP control. (B and C) KEGG pathway analysis for increased (B) and decreased (C) H3K4me3 level. ‘P’: Benjamini-normalized P value.


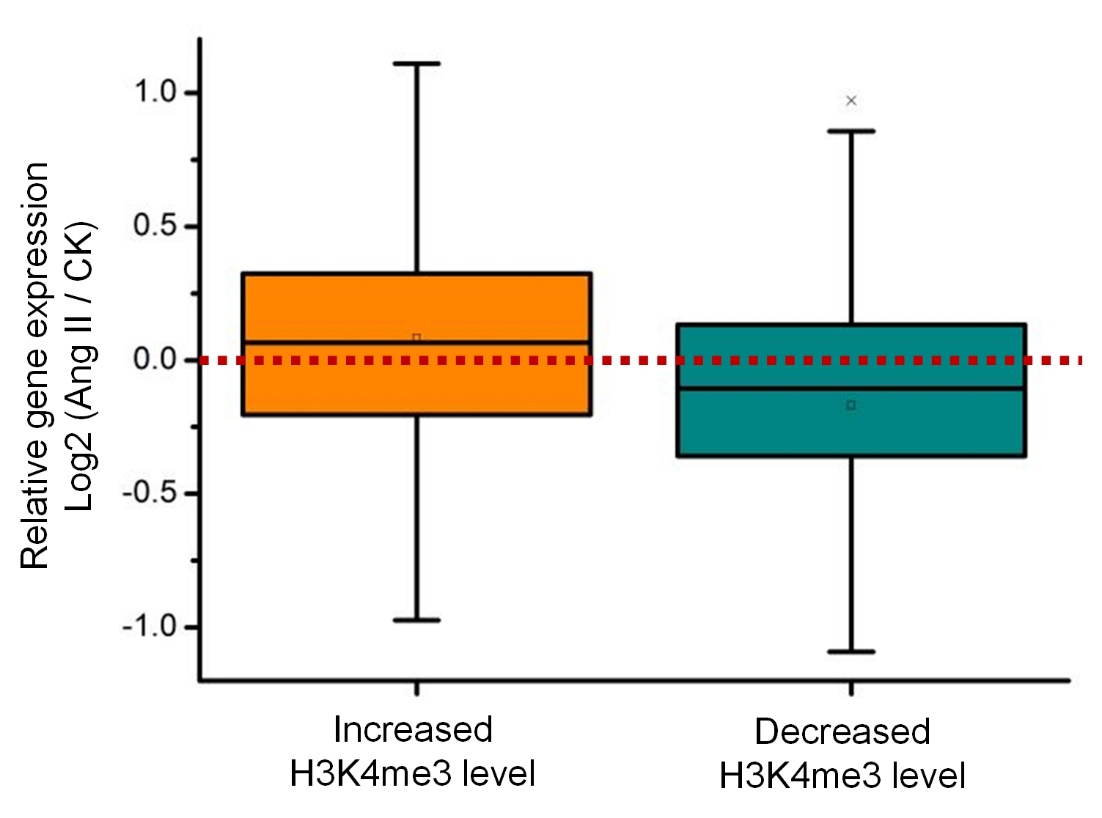


**Supplemental Figure 7.** Boxplot for comparison between gene expression (Y axis) and H3K4me3 level (X axis). Rad dashed line represents unchanged mRNA level between Ang II-treated cells and control (CK) ones.


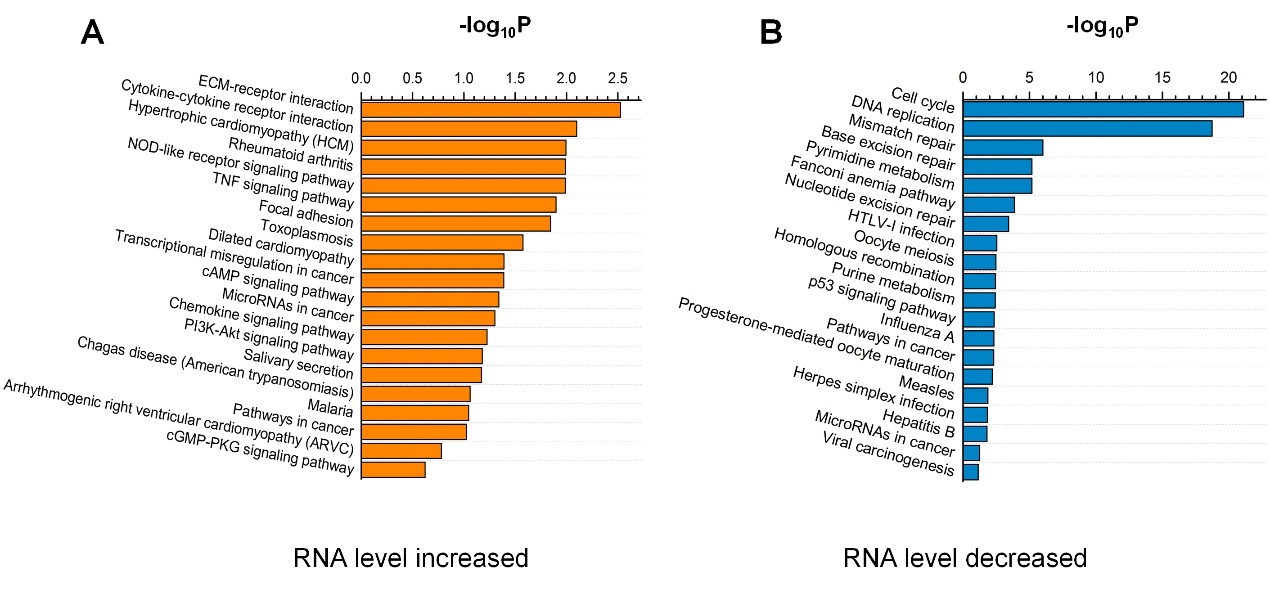


**Supplemental Figure 8.** KEGG analysis for differentially expressed genes in Ang II-treated RAEC cells. Upregulated (A) and downregulated (B) genes were determined by RNA-seq. ‘P’: Benjamini-normalized P value.


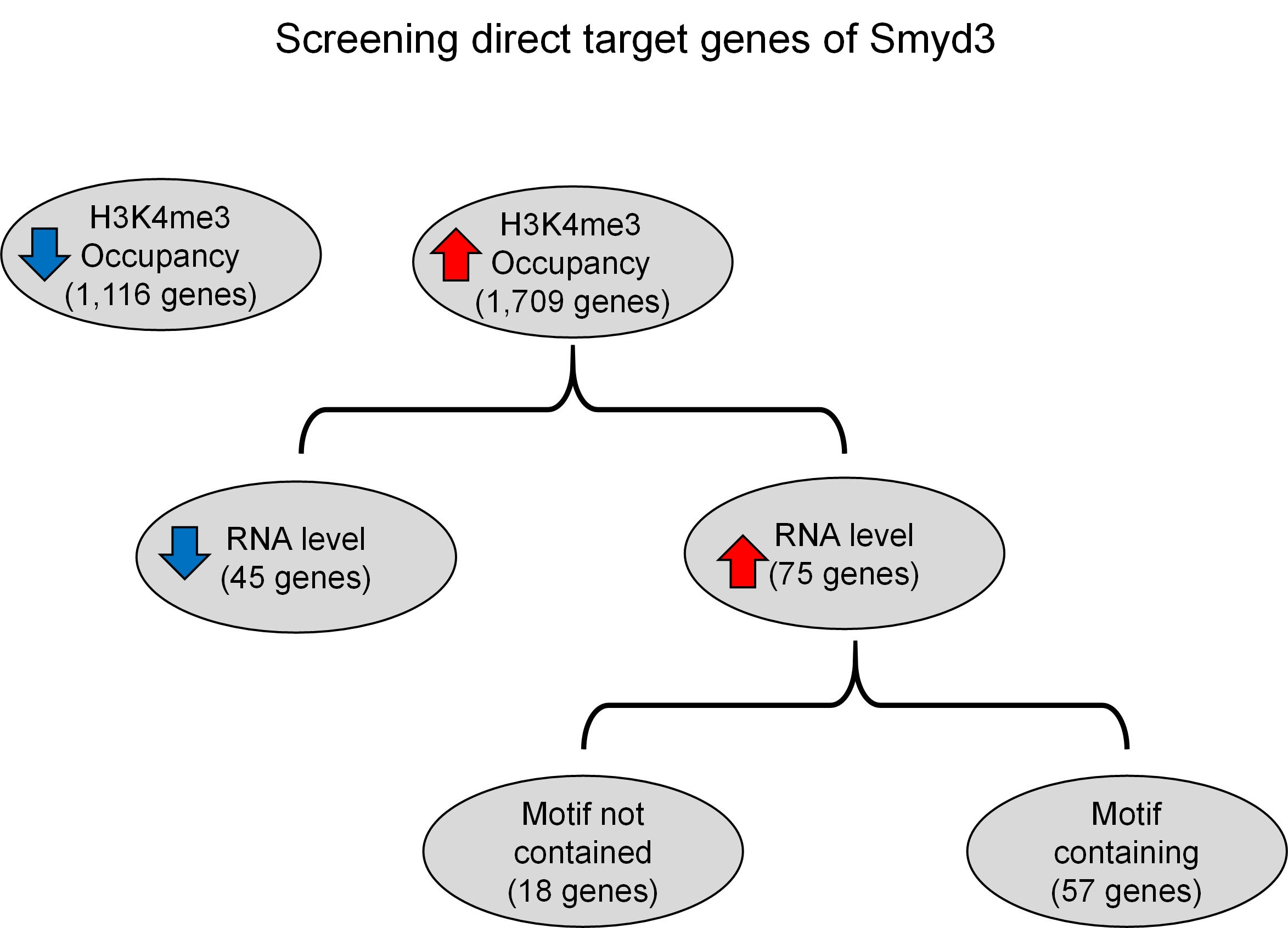


**Supplemental Figure 9.** Diagrammatic sketch showing the strategy to screen for direct target genes of Smyd3 with the following three criteria: 1) with increased H3K4me3 level near the promoter region; 2) with upregulated mRNA expression; 3) with DNA binding motif 5'-CCCTCC-3' or 5'-CCCCTC-3' of Smyd3 near the promoter region.


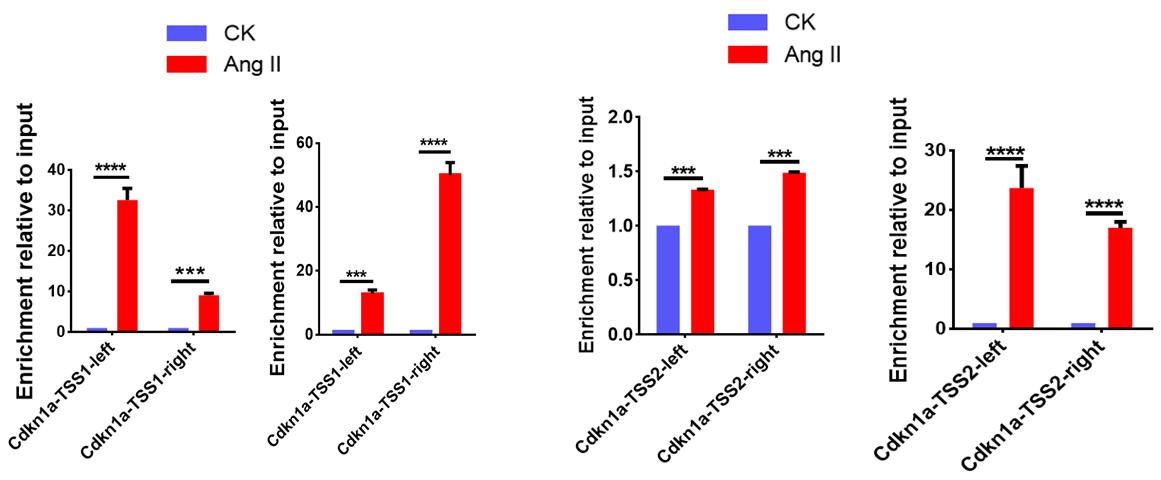


**Supplemental Figure 10.** The quantification of ChIP-PCR in Figure 4B. ^***^*p* <0.001, ^****^*p* <0.0001, all data were shown from at least three different replicates.


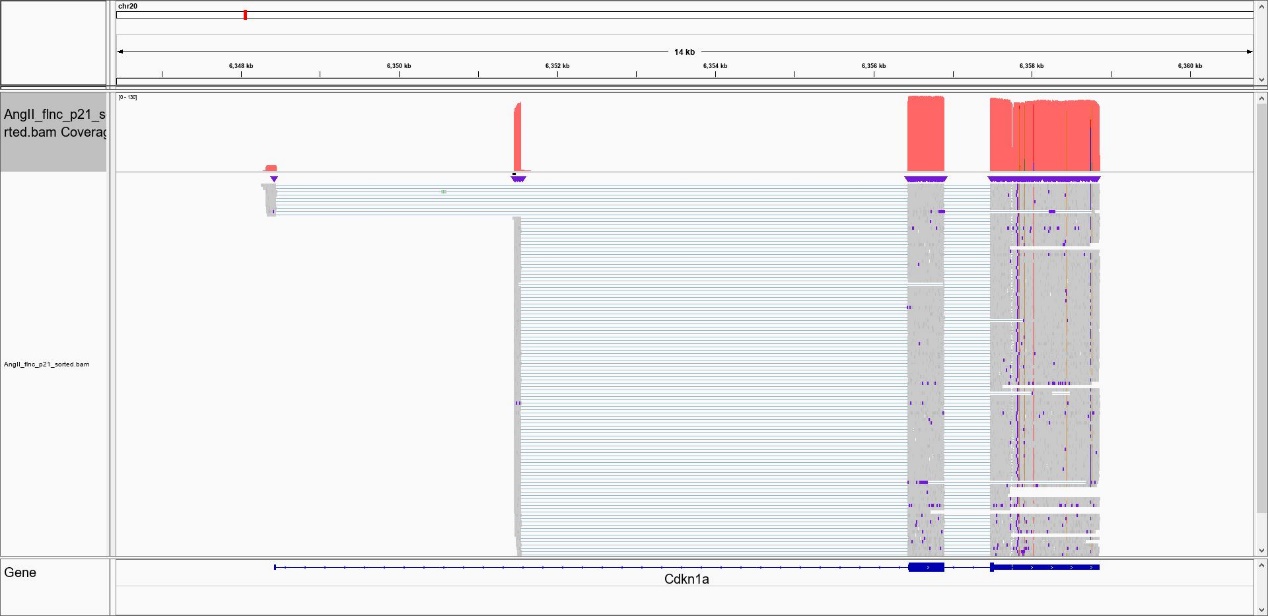

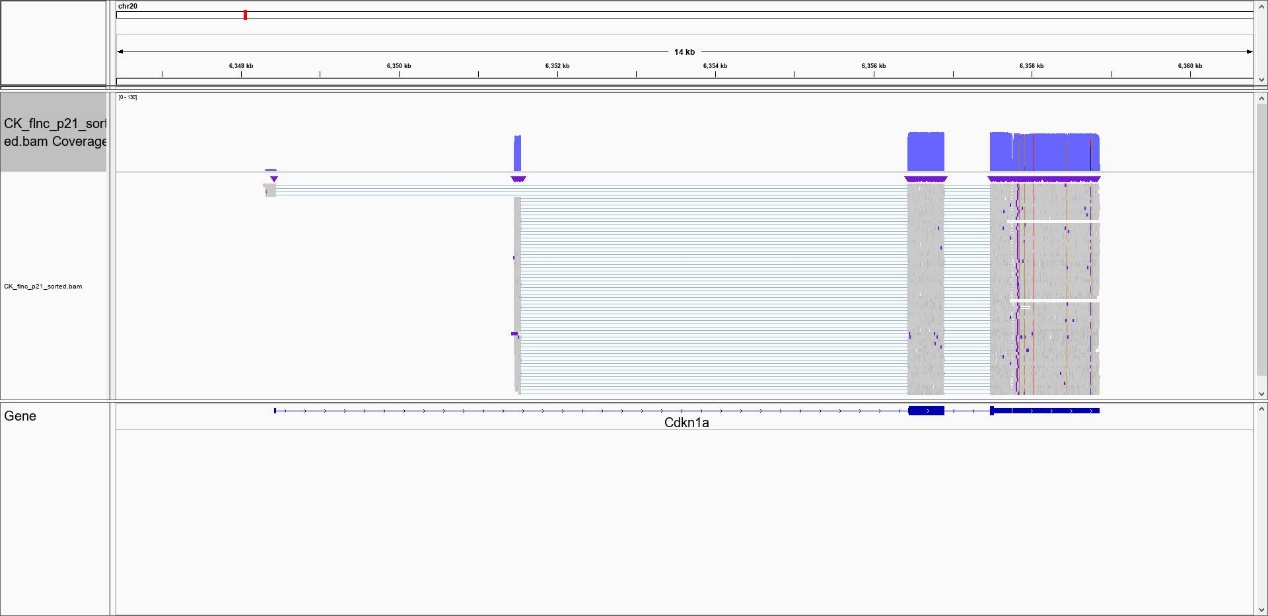


**Supplemental Figure 11.** Full-length RNA-seq (ISO-seq) tracks showing the expression of p21 transcripts derived TSS1 and TSS2. Both control (up panel) and Ang II-treated (bottom panel) cells with normalized ISO-seq tracks were shown.

**
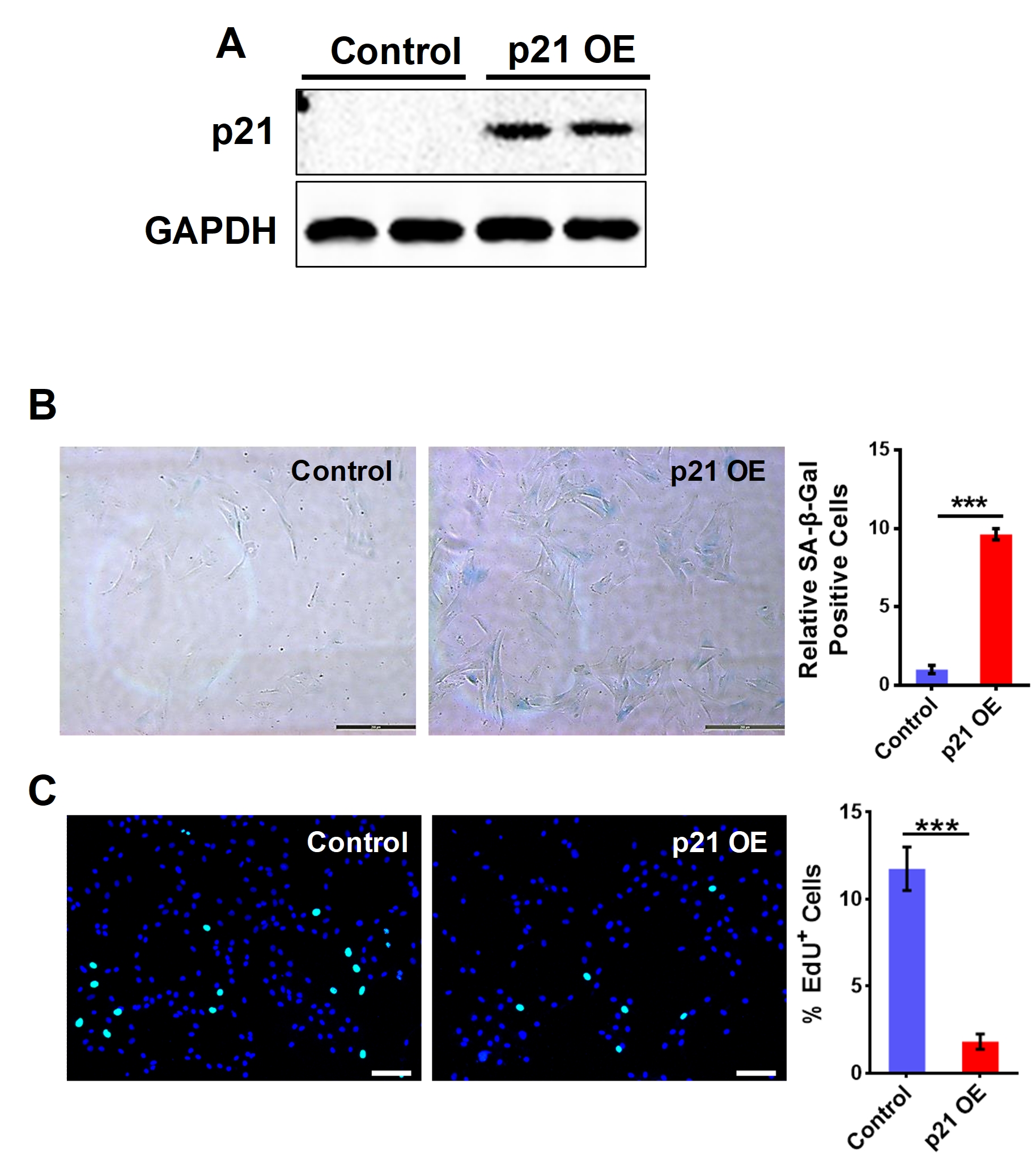
**

**Supplemental Figure 12.** p21 overexpression induces senescence in RAECs. (A) Western blot confirms p21 overexpression (p21 OE) compared to control RAEC cells. GAPDH serves as the loading control. SA-β-Gal staining with the percentage of the SA-β-Gal positive cells (B) and EdU incorporation assay with the quantification of percent EdU^+^ cells (C) in control and p21 overexpressed cells, shown as mean ± S.E.M., ^***^*p* < 0.001, (n ≥ 3).


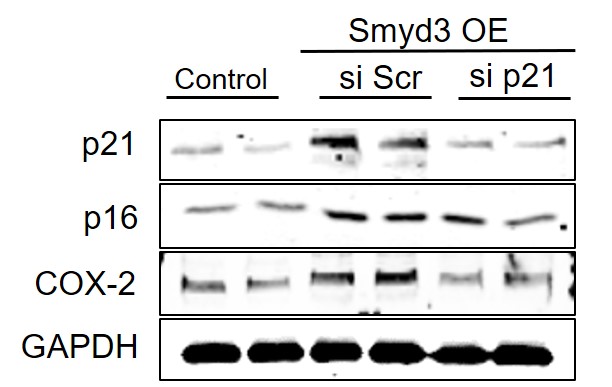

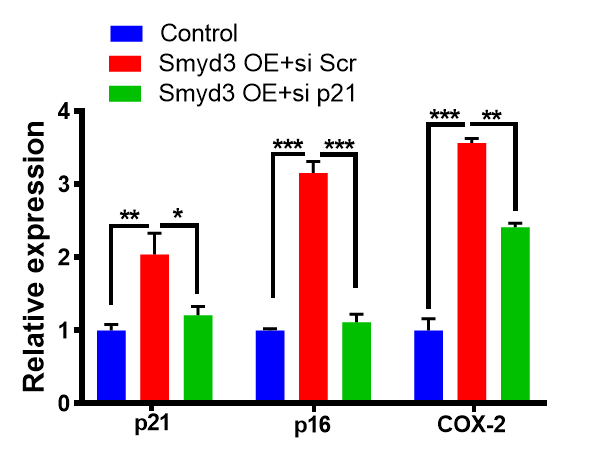


**Supplemental Figure 13**. p21 knockdown reverses vascular senescence induced by Smyd3 overexpression (Smyd3 OE) in RAECs, as evaluated by senescence related markers, p21 and COX-2. si Scr represents scramble siRNA as the negative control. Protein level of p21, p16 and COX-2 were determined by Western blotting. GAPDH serves as internal control. Left panel shows two representative replicates of Western blot image. Right panel shows quantitative statistics of Western blot. Data shown are the mean ± S.E.M. from three independent experiments, ^*^*p* < 0.05, ^**^*p* < 0.01, ^***^*p* < 0.001, t-test.


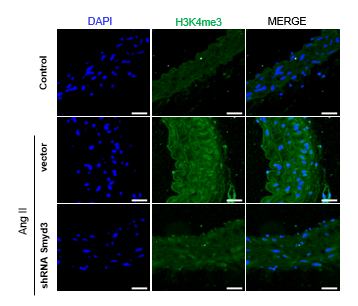


**Supplemental Figure 14**. The immunofluorescence staining of H3K4me3 in aortic great vessels of Smyd3 knockdown (shRNA Smyd3) mice and control (vector) mice with or without Ang II infusion. Visualization was realized with microscopes at 200× magnification.


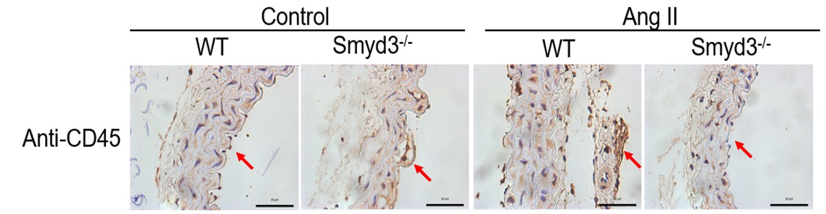

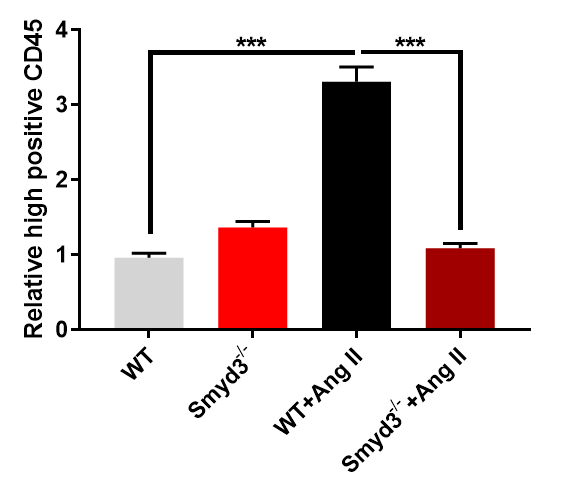


**Supplemental Figure 15**. Smyd3 deficiency attenuates Ang II-induced leukocytes infiltration. Infiltration of leukocytes around and within the vessel walls of Ang II-infusion mice was determined by immunohistochemistry with CD45 antibody (Top panel, red arrows indicate the endothelial layer). Bottom panel shows the corresponding quantitative analysis. ^***^*p* < 0.001, n ≥ 3, *t*-test.


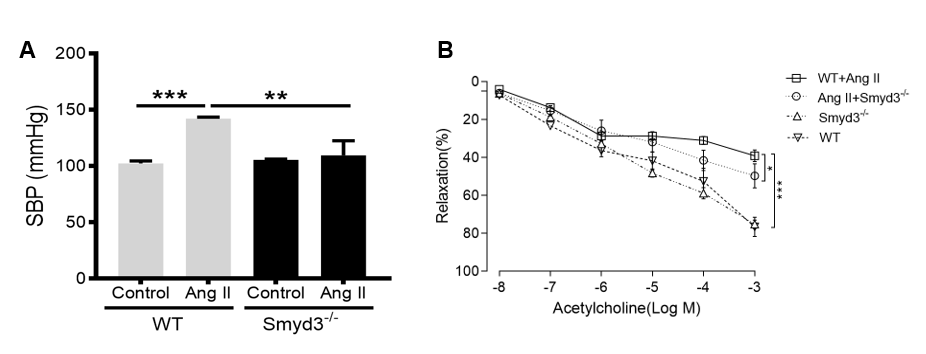
**Supplemental Figure 16.** Smyd3 deficiency decreases systolic blood pressure and improves the ACh-induced EDR of the aortic artery in Ang II-infusion mice. (A) Blood pressure was measured by tail-cuff in WT and Smyd3^-/-^ mice at the end of 28 days of Ang II infusion. ^**^*p* < 0.01 ^***^*p* < 0.001, t-test; all data were derived from at least 3 separate trials with n = 5/group. (B) ACh-induced vasorelaxation was assayed in descending aortic arteries using an organ chamber. Representative ACh-induced EDR of the aortic artery from WT or Smyd3^-/-^ mice treated with Ang II-infusion. n = 4 / group, ^*^*p* < 0.05, ^***^*p* < 0.001; SBP: systolic blood pressure; Ach: acetylcholine; EDR: endothelium dependent vasorelaxation.


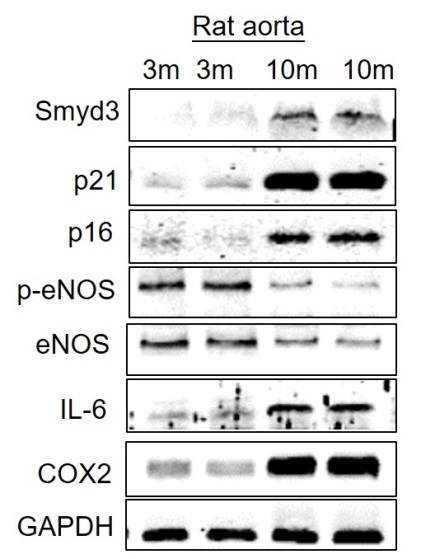

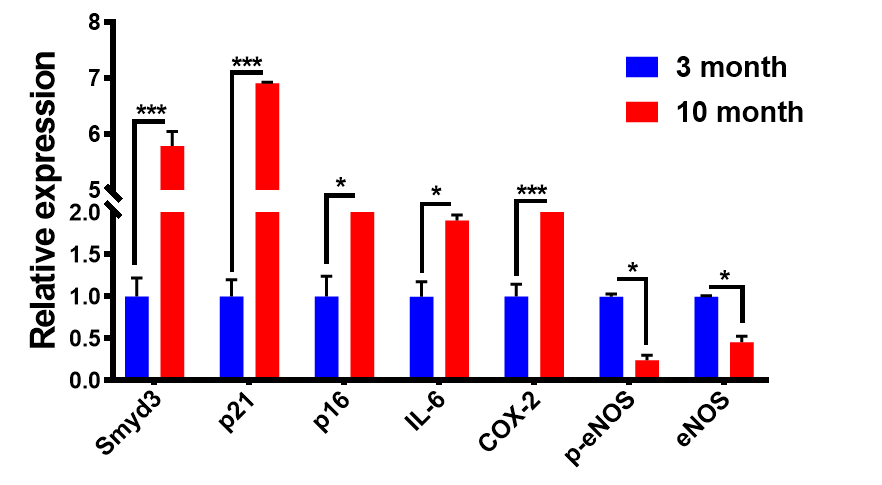


**Supplemental Figure 17.** Smyd3 is increased in aortic great vessels of relatively aged rats (10 months). The expression of Smyd3, p21, p16, COX-2, IL-6, p-eNOS and eNOS in young (3 month) and relatively aged rats (10 months) was evaluated by Western blot. GAPDH serves as the internal control. Left panel shows representative blot images of two replicates. Right panel shows quantitative statistics of Western blot. n = 3 / group, ^*^*p* < 0.05, ^***^*p* < 0.001.


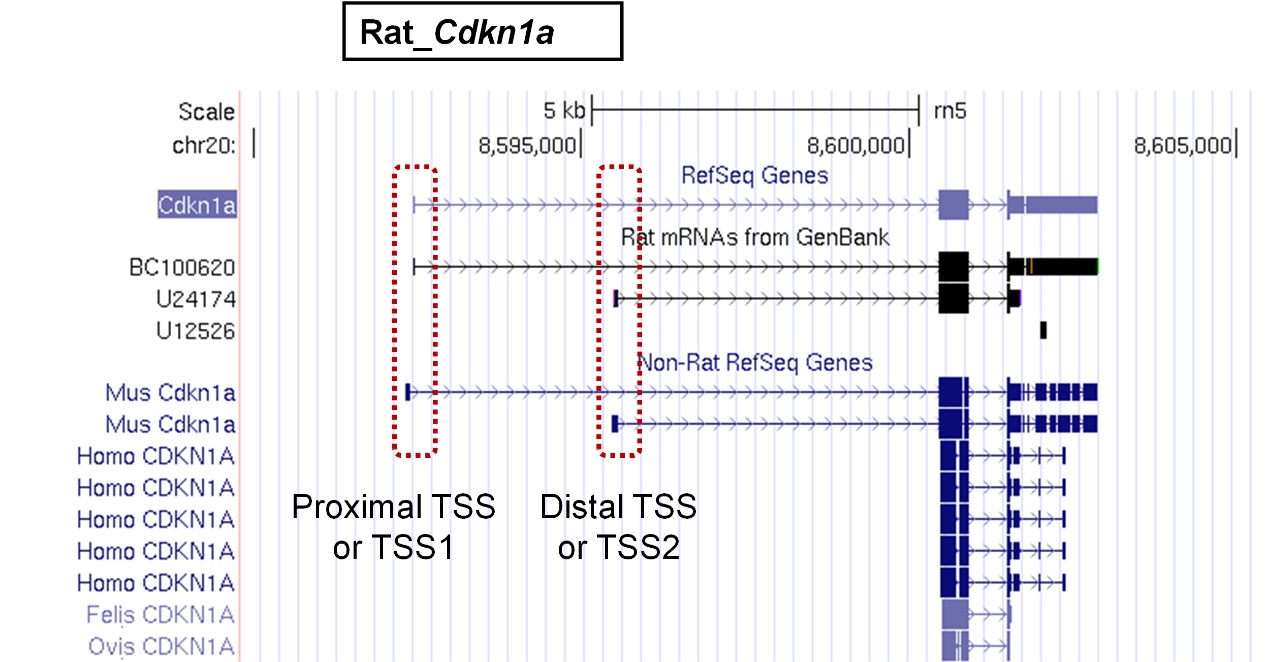


**Supplemental Figure 18.** Distal TSS of rat *Cdkn1a* was supported by mRNA from GenBank (U24174). Three gene annotation systems including RefSeq Genes, Rat mRNAs from GenBank, and Non-Rat RefSeq genes syntenic to rat *Cdkn1a* locus were visualized on UCSC genome browser.


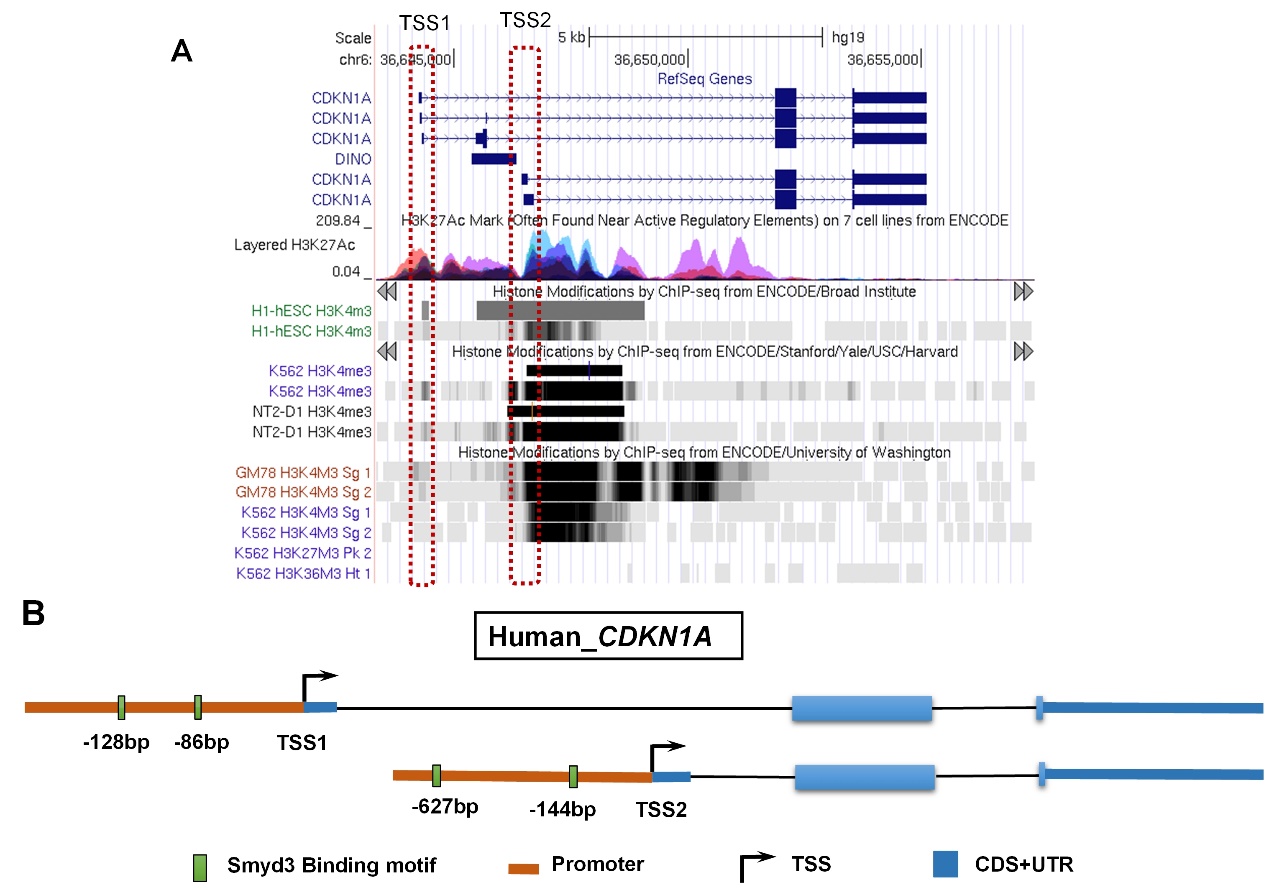


**Supplemental Figure 19.** Smyd3 binding motif exits in both promoter regions of human *CDKN1A*. (A) UCSC genome browser tracks show RefSeq Gene annotation and histone modifications including H3K27Ac and H3K4me3 in multiple human cells based on public data. Red dashed rectangles highlight the two annotated TSSs of *CDKN1A* gene. (B) Both promoters related to TSS1 and TSS2 contain Smyd3 binding motifs (green rectangles) in human *CDKN1A* gene. The numbers below the motif denote the distance (bp) upstream the TSS.


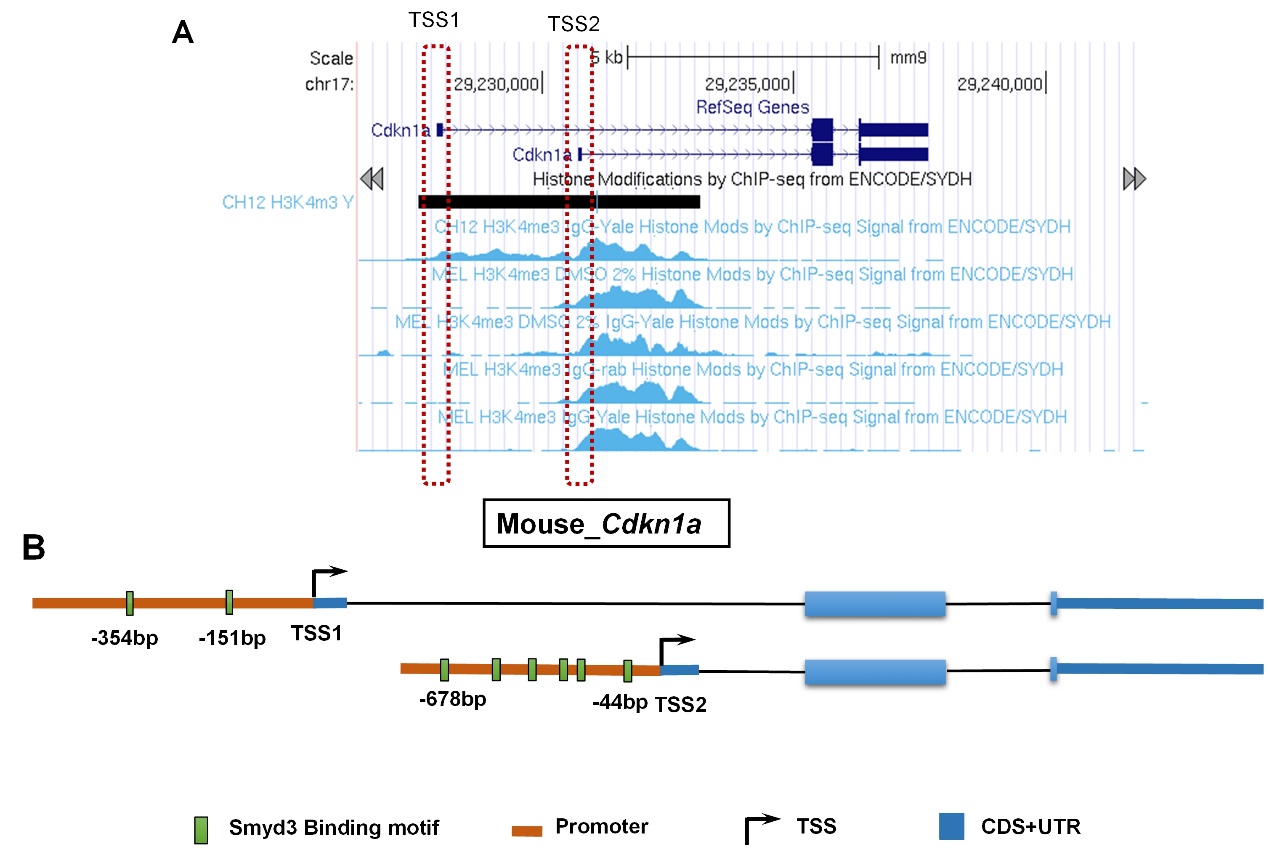


**Supplemental Figure 20.** Smyd3 binding motif exits in both two promoter regions of mouse *Cdkn1a*. (A) UCSC genome browser tracks show RefSeq Gene annotation and H3K4me3 ChIP-seq based on public data from ENCODE. Red dashed rectangles highlight the two annotated TSSs of mouse *CDKN1a* gene. (B) Both promoters related to TSS1 and TSS2 contain Smyd3 binding motifs (green rectangles) in mouse *Cdkn1a* gene. The numbers below the motif denote the distance (bp) upstream the TSS.


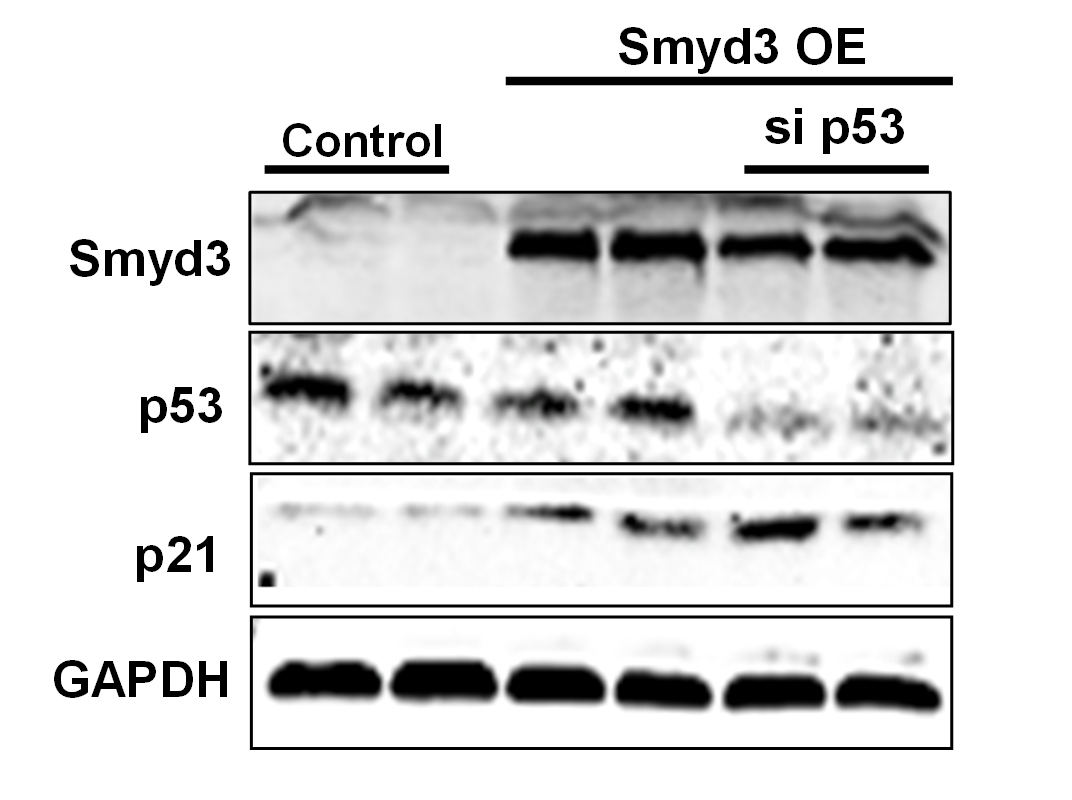


**Supplemental Figure 21.** Knockdown of p53 did not block the upregulation of p21 in Smyd3-overexpressed cells. Protein expression levels of Smyd3, p53, p21 and GAPDH in Smyd3-overexpressed (OE) RAECs, p53-knockdown (si p53) RAECs, and control RAECs were evaluated by Western blot.

**Supplemental Tables**

**Supplemental Table 1. Primers used for qRT-PCR validation.**

| Gene name | Primer name | Primer sequence (5´ to 3´) |
| --- | --- | --- |
| Cdkn1a | Rat_Cdkn1a_ F | TGCCCAAGATCTACCTGAGC |
|  | Rat_Cdkn1a_R | GTTTTCGGCCCTGAGATGTC |
| Smyd3 | Mouse_Smyd3_ F | GTGCGGGAAATTGAAGCAGG |
|  | Mouse_Smyd3_ R | CTCGTCACCCGTTAGCATGT |
| Vcam1 | Mouse_Vcam1_ F | GCTGCGAGTCACCATTGTTC |
|  | Mouse_ Vcam1_R | ACTTCGTTCCAGCTTCCCAG |

**Supplemental Table 2. Primers used for ChIP-PCR validation**

| Gene name | Primer name | | | Primer sequence (5´ to 3´) |
| --- | --- | --- | --- | --- |
| Cdkn1a | Rat_Cdkn1a_TSS1_upstream_F | | | GCTAACACGGAGCCTTCCTT |
|  | Rat_Cdkn1a_TSS1_upstream_R | | | TCCAGGGCTGAACAAGTCAA |
| Cdkn1a | Rat_Cdkn1a_TSS1_downstream_F | | | GACTGGATGGTTCAGGAGCT |
|  | Rat_Cdkn1a_TSS1_downstream_R | | | TGGAAATCTGGGAAGGGCTT |
| Cdkn1a | Rat_Cdkn1a_TSS2_1_F | | | GTGTCCCCACAAGTGTTCGT |
|  | Rat_Cdkn1a_TSS2_1_R | | | GAGTCCGCAACATAGCCTGA |
| Cdkn1a | Rat_Cdkn1a_TSS2_2_F | | | GCGAGGAGGTGACTCATTGT |
|  | Rat_Cdkn1a_TSS2_2_R | | | CCCGCCAAATAGGTCACTGT |
| Smyd3 | Rat_Smyd3_-1755bp_F | | GACGCAGACTCGTGAAGGAA | |
|  | Rat_Smyd3_-1755bp_R | ACCTCTCTTTTAGCTCACCACC | | |
| Smyd3 | Rat_Smyd3_-198bp_F | | | TTCCAGAACTGAGGCTGAGG |
|  | Rat_Smyd3_-198bp_R | | | GCTAGGGGTAGTGTGGTGTA |

**Supplemental Table 3. List for 57 potential direct target genes by Smyd3.**

| # | Gene ID | Gene Name | Gene_Description |
| --- | --- | --- | --- |
| 1 | NM_001002830 | Rasl11b | RAS-like family 11 member B |
| 2 | NM_001004249 | Tmed3 | transmembrane p24 trafficking protein 3 |
| 3 | NM_001004269 | Jam3 | junctional adhesion molecule 3 |
| 4 | NM_001007612 | Ccl7 | C-C motif chemokine ligand 7 |
| 5 | NM_001008349 | Nfkb2 | nuclear factor kappa B subunit 2 |
| 6 | NM_001008562 | Lmcd1 | LIM and cysteine-rich domains 1 |
| 7 | NM_001008694 | Rcn3 | reticulocalbin 3 |
| 8 | NM_001011942 | Cnnm2 | cyclin and CBS domain divalent metal cation transport mediator 2 |
| 9 | NM_001024745 | Slc39a6 | solute carrier family 39 member 6 |
| 10 | NM_001077589 | Rgs16 | regulator of G-protein signaling 16 |
| 11 | NM_001100535 | Col15a1 | collagen type XV alpha 1 chain |
| 12 | NM_001106420 | Adamts12 | ADAM metallopeptidase with thrombospondin type 1 motif, 12 |
| 13 | NM_001106780 | Ntn4 | netrin 4 |
| 14 | NM_001107816 | Tor4a | torsin family 4, member A |
| 15 | NM_001108142 | Sidt2 | SID1 transmembrane family, member 2 |
| 16 | NM_001108303 | Sdk2 | sidekick cell adhesion molecule 2 |
| 17 | NM_001108613 | Slc17a9 | solute carrier family 17 member 9 |
| 18 | NM_001108859 | Irf6 | interferon regulatory factor 6 |
| 19 | NM_001109119 | Tubb2a | tubulin, beta 2A class IIa |
| 20 | NM_001109236 | Nod1 | nucleotide-binding oligomerization domain containing 1 |
| 21 | NM_001109616 | Fam219a | family with sequence similarity 219, member A |
| 22 | NM_001130542 | Sntb1 | syntrophin, beta 1 |
| 23 | NM_001130548 | Col14a1 | collagen type XIV alpha 1 chain |
| 24 | NM_001135158 | Myh1 | myosin heavy chain 1 |
| 25 | NM_001135799 | Bmper | BMP-binding endothelial regulator |
| 26 | NM_001137561 | Rnasek | ribonuclease K |
| 27 | NM_001170480 | Phlda2 | pleckstrin homology-like domain, family A, member 2 |
| 28 | NM_001191763 | Mycl | MYCL proto-oncogene, bHLH transcription factor |
| 29 | NM_001191986 | Cyp4f17 | cytochrome P450, family 4, subfamily f, polypeptide 17 |
| 30 | NM_001244933 | Nfatc1 | nuclear factor of activated T-cells 1 |
| 31 | NM_001270701 | Kcnn4 | potassium calcium-activated channel subfamily N member 4 |
| 32 | NM_001271371 | Anks1b | ankyrin repeat and sterile alpha motif domain containing 1B |
| 33 | NM_001304816 | Peg3 | paternally expressed 3 |
| 34 | NM_012801 | Pdgfa | platelet derived growth factor subunit A |
| 35 | NM_012867 | Ninj1 | ninjurin 1 |
| 36 | NM_013115 | Ptgfr | prostaglandin F receptor |
| 37 | NM_017204 | Map6 | microtubule-associated protein 6 |
| 38 | NM_019340 | Rgs3 | regulator of G-protein signaling 3 |
| 39 | NM_022618 | Akap6 | A-kinase anchoring protein 6 |
| 40 | NM_030833 | Ifitm2 | interferon induced transmembrane protein 2 |
| 41 | NM_030842 | Itga7 | integrin subunit alpha 7 |
| 42 | NM_030868 | Ccn3 | cellular communication network factor 3 |
| 43 | NM_031667 | Syt11 | synaptotagmin 11 |
| 44 | NM_031753 | Alcam | activated leukocyte cell adhesion molecule |
| 45 | NM_031798 | Slc12a2 | solute carrier family 12 member 2 |
| 46 | NM_053535 | Enpp1 | ectonucleotide pyrophosphatase/phosphodiesterase 1 |
| 47 | NM_053568 | Pcyt2 | phosphate cytidylyltransferase 2, ethanolamine |
| 48 | NM_053585 | Madd | MAP-kinase activating death domain |
| 49 | NM_053629 | Fstl3 | follistatin like 3 |
| 50 | NM_053727 | Nfil3 | nuclear factor, interleukin 3 regulated |
| 51 | NM_080403 | Sox9 | SRY-box transcription factor 9 |
| 52 | NM_080782 | Cdkn1a | cyclin-dependent kinase inhibitor 1A |
| 53 | NM_133425 | Ppp1r14c | protein phosphatase 1, regulatory (inhibitor) subunit 14c |
| 54 | NM_172017 | Yif1a | Yip1 interacting factor homolog A, membrane trafficking protein |
| 55 | NM_172042 | Kcnk2 | potassium two pore domain channel subfamily K member 2 |
| 56 | NM_182667 | Myocd | myocardin |
| 57 | NM_199407 | Unc5c | unc-5 netrin receptor C |

**Supplemental References**

Barski, A., Cuddapah, S., Cui, K., Roh, T. Y., Schones, D. E., Wang, Z., . . . Zhao, K. (2007). High-resolution profiling of histone methylations in the human genome. *Cell, 129*(4), 823-837. doi:10.1016/j.cell.2007.05.009

Cervenka, L., Wang, C. T., Mitchell, K. D., & Navar, L. G. (1999). Proximal tubular angiotensin II levels and renal functional responses to AT1 receptor blockade in nonclipped kidneys of Goldblatt hypertensive rats. *Hypertension, 33*(1), 102-107.

Huang da, W., Sherman, B. T., & Lempicki, R. A. (2009). Systematic and integrative analysis of large gene lists using DAVID bioinformatics resources. *Nat Protoc, 4*(1), 44-57. doi:10.1038/nprot.2008.211

Itahana, K., Campisi, J., & Dimri, G. P. (2007). Methods to detect biomarkers of cellular senescence: the senescence-associated beta-galactosidase assay. *Methods in Molecular Biology, 371*, 21-31.

Langmead, B., Trapnell, C., Pop, M., & Salzberg, S. L. (2009). Ultrafast and memory-efficient alignment of short DNA sequences to the human genome. *Genome Biol, 10*(3), R25. doi:10.1186/gb-2009-10-3-r25

Li, H., & Durbin, R. (2009). Fast and accurate short read alignment with Burrows-Wheeler transform. *Bioinformatics, 25*(14), 1754-1760. doi:10.1093/bioinformatics/btp324

Li, H., Handsaker, B., Wysoker, A., Fennell, T., Ruan, J., Homer, N., . . . Genome Project Data Processing, S. (2009). The Sequence Alignment/Map format and SAMtools. *Bioinformatics, 25*(16), 2078-2079. doi:10.1093/bioinformatics/btp352

Parkhomchuk, D., Borodina, T., Amstislavskiy, V., Banaru, M., Hallen, L., Krobitsch, S., . . . Soldatov, A. (2009). Transcriptome analysis by strand-specific sequencing of complementary DNA. *Nucleic Acids Res, 37*(18), e123. doi:10.1093/nar/gkp596

Trapnell, C., Pachter, L., & Salzberg, S. L. (2009). TopHat: discovering splice junctions with RNA-Seq. *Bioinformatics, 25*(9), 1105-1111. doi:10.1093/bioinformatics/btp120

Trapnell, C., Williams, B. A., Pertea, G., Mortazavi, A., Kwan, G., van Baren, M. J., . . . Pachter, L. (2010). Transcript assembly and quantification by RNA-Seq reveals unannotated transcripts and isoform switching during cell differentiation. *Nat Biotechnol, 28*(5), 511-515. doi:10.1038/nbt.1621

Wang, Z., Zang, C., Rosenfeld, J. A., Schones, D. E., Barski, A., Cuddapah, S., . . . Zhao, K. (2008). Combinatorial patterns of histone acetylations and methylations in the human genome. *Nat Genet, 40*(7), 897-903. doi:10.1038/ng.154

Yang, D., Xiao, C., Long, F., Su, Z., Jia, W., Qin, M., . . . Zhu, Y. (2018). HDAC4 regulates vascular inflammation via activation of autophagy. *Cardiovascular Research*. doi:10.1093/cvr/cvy051

Zhang, Y., Liu, T., Meyer, C. A., Eeckhoute, J., Johnson, D. S., Bernstein, B. E., . . . Liu, X. S. (2008). Model-based analysis of ChIP-Seq (MACS). *Genome Biol, 9*(9), R137. doi:10.1186/gb-2008-9-9-r137
